# Bromination: An Alternative Strategy for Non‐Fullerene Small Molecule Acceptors

**DOI:** 10.1002/advs.201903784

**Published:** 2020-02-28

**Authors:** Huan Wang, Tao Liu, Jiadong Zhou, Daize Mo, Liang Han, Hanjian Lai, Hui Chen, Nan Zheng, Yulin Zhu, Zengqi Xie, Feng He

**Affiliations:** ^1^ Shenzhen Grubbs Institute and Department of Chemistry Southern University of Science and Technology Shenzhen 518055 China; ^2^ Faculty of Health Sciences University of Macau Macao 999078 China; ^3^ Institute of Polymer Optoelectronic Materials and Devices State Key Laboratory of Luminescent Materials and Devices South China University of Technology Guangzhou 510640 China

**Keywords:** bromination, intermolecular interactions, non‐fullerene acceptors, open‐circuit voltage, polymer solar cells

## Abstract

The concept of bromination for organic solar cells has received little attention. However, the electron withdrawing ability and noncovalent interactions of bromine are similar to those of fluorine and chlorine atoms. A tetra‐brominated non‐fullerene acceptor, designated as BTIC‐4Br, has been recently developed by introducing bromine atoms onto the end‐capping group of 2‐(3‐oxo‐2,3‐dihydro‐1H‐inden‐1‐ylidene) malononitrile and displayed a high power conversion efficiency (PCE) of 12%. To further improve its photovoltaic performance, the acceptor is optimized either by introducing a longer alkyl chain to the core or by modulating the numbers of bromine substituents. After changing each end‐group to a single bromine, the BTIC‐2Br‐*m*‐based devices exhibit an outstanding PCE of 16.11% with an elevated open‐circuit voltage of *V*_oc_ = 0.88 V, one of the highest PCEs reported among brominated non‐fullerene acceptors. This significant improvement can be attributed to the higher light harvesting efficiency, optimized morphology, and higher exciton quenching efficiencies of the di‐brominated acceptor. These results demonstrate that the substitution of bromine onto the terminal group of non‐fullerene acceptors results in high‐efficiency organic semiconductors, and promotes the use of the halogen‐substituted strategy for polymer solar cell applications.

## Graphical abstract

The bromination of non‐fullerene acceptors provides a promising alternative approach for the creation of high‐performance organic solar cells. BTIC‐2Br‐*m*‐based devices exhibit an outstanding power conversion efficiency of 16.11% with an elevated open circuit voltage of 0.88 V, representing one of the highest efficiencies in brominated non‐fullerene acceptors.

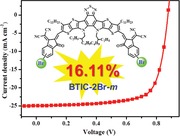

Solution‐processed bulk heterojunction (BHJ) organic solar cells (OSCs) based on the p‐type conjugated polymer donors and the n‐type semiconductor acceptors have attracted enormous attention due to the unique advantages they provide for the fabrication of low‐cost and flexible devices.^[^ 1, 2, 3, 4, 5 ^]^ In recent years, innovations in photovoltaic materials and device modifications have led to a great progress in the power conversion efficiency (PCE), increasing up to 16%, for polymer solar cells (PSCs).^[^ 6, 7, 8 ^]^ The intrinsic properties of the semiconductor materials, such as their band gaps (*E*_g_), frontier energy levels, optical absorption spectra, and processability, are crucial to achieve the desired level of device performance.^[^ 9, 10, 11, 12 ^]^ In terms of high‐efficiency acceptor materials, fullerene derivatives have dominated the field for electron acceptors with a PCE >11% in previous years.^[^ 13, 14 ^]^ However, non‐fullerene acceptors have recently emerged as promising candidates owing to their efficient light absorption in the visible region to the near‐infrared region and easily‐tunable energy levels,^[^ 15, 16, 17, 18 ^]^ which have greatly enhanced the photovoltaic performance, resulting in a PCE over 16%.

Halogenation is an effective strategy used to increase the optical absorption, refine the energy levels, and improve the molecular packing of semiconductor materials.^[^ 19, 20, 21, 22 ^]^ Numerous studies^[^ 23, 24, 25, 26, 27, 28, 29 ^]^ have reported on the use of fluorine in OSCs materials and have indicated that the small atom size of fluorine provides the highest electronegativity, leading to the modulation of the π‐electron properties.^[^ 30, 31 ^]^ More recently, chlorinated materials have emerged as a promising alternative in the field of solar energy.^[^ 32, 33, 34, 35, 36, 37 ^]^ The research has demonstrated that chlorination is more effective in down‐shifting the energy levels compared to fluorination, due to the empty 3D orbitals of the chlorine atoms, which benefit from accepting electron pairs or π‐electrons.^[^ 33, 34, 35 ^]^ Furthermore, the dipole moment of the C—Cl bond is higher than that of the C—F bond, which enhances the intramolecular charge transfer (ICT), thus broadening the spectrum of optical absorption.^[^ 35 ^]^ Chlorinated materials have also drawn attention due to their lower costs for the synthesis of target materials.^[^ 38, 39, 40, 41 ^]^ In comparison to fluorine and chlorine, very little attention has been paid to bromine in this area of research. Bromine is also a halogen atom and demonstrates a strong electronegativity and noncovalent interactions. To date, few studies have investigated the use of bromination in non‐fullerene small molecule acceptors, despite the fact that it results in a comparable or a superior photovoltaic performance compared with the fluorinated or chlorinated analogues.^[^ 22, 42 ^]^ The use of bromine as an alternative to halogen atoms is worthy of investigation for use in OSCs due to its capability for further polymerization. Zou et al. recently reported on a novel efficient fluorinated acceptor, Y6, containing a ladder‐type electron‐deficient‐core‐based central fused ring, which boosted the PCE of organic solar cells by up to 15.7%.^[^ 18 ^]^ Subsequently, Hou et al. obtained a higher PCE of over 16% by replacing the fluorine atom of the Y6 acceptor with chlorine atoms.^[^ 7 ^]^

Based on the design principles of these studies, we developed a brominated small molecular BTIC‐4Br (see **Scheme** **1**) by replacing the halogen atoms in Y6 with bromine to understand the role of the bromination in regulating solar conversion. However, BTIC‐4Br exhibited a relatively poor solubility in a chloroform solution due to a tetra‐bromine substitution, exhibiting a PCE of only 7.51% when the mixed solution was dissolved at room temperature. To overcome this issue, the dissolution temperature was increased to 50 °C, resulting in increased solubility and a PCE of 12.20%. A series of brominated acceptors were designed and synthesized by either introducing the longer alkyl chain to the core or by modulating the numbers of bromine substituents, designated as BTIC‐BO‐4Br and BTIC‐2Br‐*m*. The photovoltaic performance of the BTIC‐BO‐4Br‐ and BTIC‐2Br‐*m*‐based devices achieved an efficiency of 14.03% and 16.11%, respectively. This result indicates the great potential of bromination in promoting the photovoltaic performance of small molecular acceptors. Bromination has great potential for use in current organic solar conversion, the study of which may eventually lead to the development of more highly efficient brominated materials to boost the performance of organic solar devices.

**Scheme 1:**
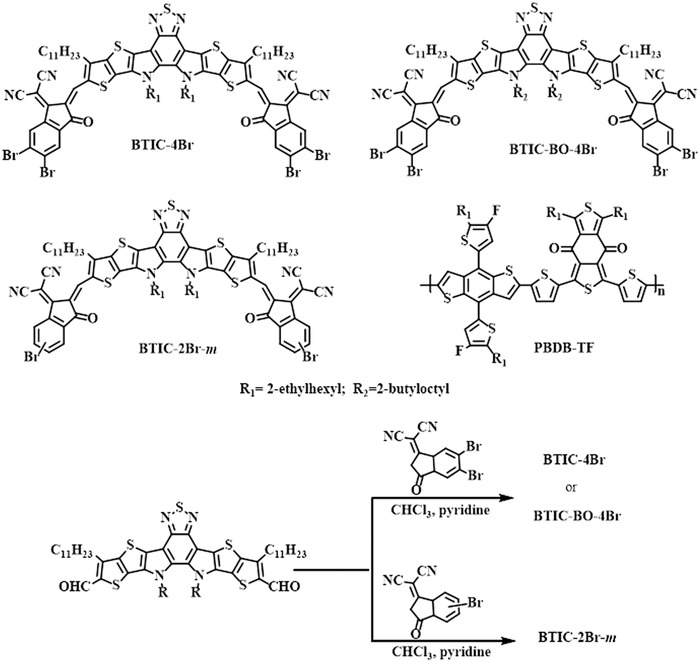
The chemical structures and the synthetic routes of BTIC‐4Br, BTIC‐BO‐4Br and BTIC‐2Br‐*m*.

The chemical structures and synthetic routes of the acceptors have been shown in Scheme 1 while details of synthesis have been provided in the Supporting Information. Polymer PBDB‐TF was used as a donor with a number‐average molecular weight (*M*_n_) of 48.0 kDa and a polydispersity index of 2.41. The target products, namely BTIC‐4Br, BTIC‐BO‐4Br, and BTIC‐2Br‐*m*, were achieved between the corresponding ending‐groups and the dialdehyde intermediate using the Knoevenagel condensation reaction. The small molecular acceptors were purified using column chromatography on silica gel with chloroform as the eluent, coupled with a preparative high‐performance liquid chromatography. With four bromine atoms on the end‐groups, the corresponding acceptor, BTIC‐4Br, showed a relatively limited solubility in chlorobenzene and chloroform. In comparison, the other two small molecules, namely BTIC‐BO‐4Br, with longer branched side chains, and BTIC‐2Br‐*m*, with fewer bromine substitutes, showed a better solubility in common solvents. Additionally, a single‐crystal of BTIC‐BO‐4Br was successfully obtained using the slow diffusion growth method (Supporting Information) to investigate the intermolecular interactions and molecular packing (Figure S1–S3, Supporting Information). As shown in Figure S1, Supporting Information, the distance of S—O was 2.67 Å in a single BTIC‐BO‐4Br molecule, endowing this arc‐like acceptor with a very good planarity in terms of its single molecular configuration. Despite the large atomic radius of the bromine (Br) atom, BTIC‐BO‐4Br exhibited short π–π distances (3.43–3.48 Å) between the adjacent molecules (Figure S2c, Supporting Information), This was due to the multiple inter‐locked Br—S and Br—π interactions, with distances of 3.77, 4.44, 3.81, and 3.68 Å, respectively. Meanwhile, these interactions allowed BTIC‐BO‐4Br to present a kind of spiral‐like microstructures consisting of six molecules in four layers in a vertical direction, resulting in a closed spiral ring (Figure S2c, Supporting Information). This eventually formed a 3D interpenetrating network, which may be beneficial for the transportation of charge carriers in the acceptor (Figure S3, Supporting Information).

To evaluate the optical properties of the brominated acceptors, the ultraviolet‐visible (UV–vis) absorption spectra of the three acceptors in a chloroform solution and thin films were recorded. BTIC‐4Br, BTIC‐BO‐4Br, and BTIC‐2Br‐*m* in the chloroform solution (10^−5^ mol L^−1^) displayed a similar absorption in a wavelength range of 700–800 nm, with a maximum absorption peak at 746, 747, and 734 nm, respectively (**Figure** **1**a). In addition, the extinction coefficients of BTIC‐4Br, BTIC‐BO‐4Br, and BTIC‐2Br‐*m* in the solution were measured to be 1.55 × 10^5^, 1.76 × 10^5^, and 1.65 × 10^5^ L mol^−1^ cm^−1^, respectively. As shown in Figure 1b, the absorption peaks of BTIC‐4Br, BTIC‐BO‐4Br, and BTIC‐2Br‐*m* in the thin film were 825, 823, and 820 nm, respectively. Compared to the absorption spectra recorded in solution, the bathochromic shifts of 79, 76, and 86 nm in the film state for BTIC‐4Br, BTIC‐BO‐4Br, and BTIC‐2Br‐*m*, respectively, indicated the strong molecular aggregation and the π–π interactions of the acceptors in the film states. Even BTIC‐2Br‐*m* in solution showed the most blue‐shifted absorption and exhibited the biggest red‐shift from solution to film. It is noteworthy that the extinction coefficients of BTIC‐2Br‐*m* and BTIC‐BO‐4Br in the film state were about 8.71 × 10^4^ and 7.82 × 10^4^ cm^−1^, respectively, which were both greater than that of the BTIC‐4Br film (5.01 × 10^4^ cm^−1^). In addition, when mixed with the donor PBDB‐TF (Figure 1c), the blend films based on the three small molecules exhibited a similar extinction coefficient of about 6.70 × 10^4^ cm^−1^ at 628 nm, which was mainly the result of the donor PBDB‐TF. Additionally, the blend films based on BTIC‐BO‐4Br and BTIC‐2Br‐*m* both exhibited stronger extinction coefficients, 6.97 × 10^4^ and 7.26 × 10^4^ cm^−1^, respectively, than BTIC‐4Br, with 3.67 × 10^4^ cm^−1^, in the wavelength range of 750–850 nm. This has been ascribed to the lower extinction coefficient of BTIC‐4Br. This stronger absorption is beneficial for enhancing the light harvesting efficiencies and leads to an improved *J*_sc_ in solar devices.^[^ 12, 43 ^]^

**Figure 1:**
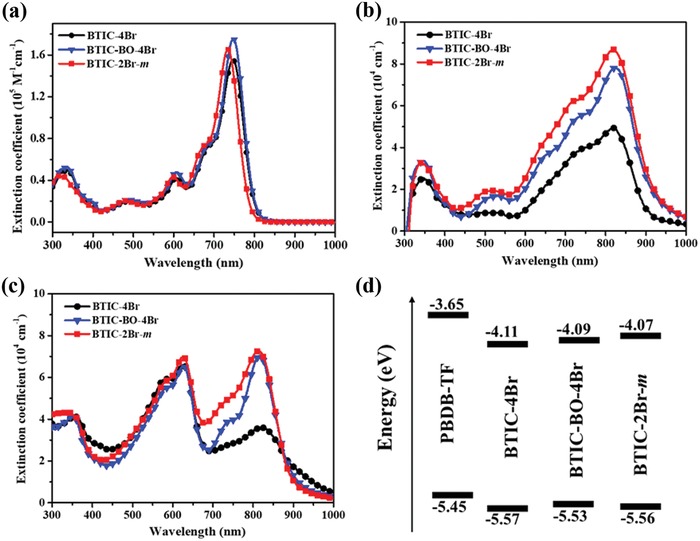
Calculated absorption spectra of BTIC‐4Br, BTIC‐BO‐4Br and BTIC‐2Br‐*m* a) in solution, b) in films, c) in the blend films with donor PBDB‐TF. d) Energy level alignments of PBDB‐TF, BTIC‐4Br, BTIC‐BO‐4Br and BTIC‐2Br‐*m*.

Cyclic voltammetry (CV) was used to determine and evaluate the electrochemical properties of the three small molecules. The electrochemical parameters have been summarized in **Table** **1**, and the corresponding oxidation and redox curves have been shown in Figure S4, Supporting Information. The LUMO levels of BTIC‐4Br, BTIC‐BO‐4Br, and BTIC‐2Br‐*m* were −4.11, ‒4.09, and ‒4.07 eV, while the HOMO levels were ‒5.57, ‒5.53, and ‒5.56 eV, respectively. These results indicated that with the successive increase of bromine substitution, the LUMO level of BTIC‐4Br decreased compared to that of BTIC‐2Br‐*m* due to the electron‐withdrawing effect of the bromine atoms.^[^ 44, 45 ^]^ After lengthening the alkyl chain slightly, the LUMO levels of BTIC‐BO‐4Br only increased by about 0.02 V, which indicated that the open‐circuit voltage (*V*_oc_) of the BTIC‐BO‐4Br‐based devices was very close to that of BTIC‐4Br‐based devices. However, BTIC‐2Br‐*m* showed the highest *V*_oc_ among the three acceptors. Moreover, as shown in Figure 1d, the small HOMO offsets between PBDB‐TF and the three brominated acceptors (0.08–0.12 eV) implied that a small driving force was able to support the charge separation while maintaining a low energy loss.^[^ 46, 47, 48 ^]^

**Table 1:** Optical and electronic properties of BTIC‐4Br, BTIC‐BO‐4Br and BTIC‐2Br‐*m*

Acceptor	Solution		Film		HOMO [eV]^a)^	LUMO [eV]^b)^	*E*_g_^ec^ [eV]^c)^
	λ_max_ [nm]	*e* [10^5^ L mol^−1^ cm^−1^]	λ_max_ [nm]	*e* [10^4^ cm^−1^]			
BTIC‐4Br	746	1.55	825	5.01	−5.57	−4.11	1.46
BTIC‐BO‐4Br	747	1.76	823	7.82	−5.53	−4.09	1.44
BTIC‐2Br‐*m*	734	1.65	820	8.71	−5.56	−4.07	1.49

^a)^ HOMO = −(*E*_ox_ + 4.8‐E_Fc/Fc+_)

^b)^ LUMO = −(*E*_red_ + 4.8‐E_Fc/Fc+_)

^c)^ *E*_g_^ec^ = LUMO‐HOMO.

To further investigate the molecular energy levels and the optimal geometries of the three small molecules, their density functional theory (DFT) was measured at the B3LYP 6–31G(d,p) level. All alkyl groups were replaced with methyl groups to simplify the calculations. In terms of the mixture of isomers BTIC‐2Br‐*m*, the two possible molecular skeletons were denoted as BTIC‐2Br‐1 and BTIC‐2Br‐2. Meanwhile, BTIC‐4Br and BTIC‐BO‐4Br exhibited the same molecular skeletons, denoted as BTIC‐4Br‐α. Figure S5, Supporting Information shows the optimized molecular geometries and the orbital distributions of the acceptors. Only small differences were found, due to their homogeneous molecular backbones. Between BTIC‐4Br to BTIC‐2Br‐*m*, the HOMO (‒5.63 eV to ‒5.56 eV/‒5.57 eV) and the LUMO (‒3.61 eV to ‒3.53 eV/‒3.52 eV) levels were upshifted, which was consistent with the results from cyclic voltammetry. These results were able to also predict a higher *V*_oc_ when applying BTIC‐2Br‐*m* as an acceptor in the OSCs. In contrast, the overall molecular dipole moments (Figure S5, Supporting Information) were 0.52 Debye, 2.94 Debye, and 2.07 Debye for BTIC‐4Br‐α, BTIC‐2Br‐1, and BTIC‐2Br‐2, respectively. This pronounced difference indicates that the bromine atoms play a crucial role in the polarization of the molecular skeleton.

To investigate the photovoltaic performance of the brominated acceptors, the devices were fabricated with an inverted structure (ITO/ZnO/active layer/MoO_3_/Ag) using the polymer PBDB‐TF as the donor in a chloroform solution. The characteristic current density‐voltage (*J*–*V*) curves and the corresponding photovoltaic parameters for the best devices have been shown in **Figure** **2**a and have been summarized in **Table** **2**. To obtain the best device performance of BTIC‐4Br, we varied the total concentration of the mixed solution and maintained the overall ratio of PBDB‐TF:BTIC‐4Br at 1:1.2 (Table S1, Supporting Information). The best PBDB‐TF:BTIC‐4Br‐based device exhibited a poor PCE of 7.51% with a moderate *V*_oc_ of 0.87 V, *J*_sc_ of 20.65 mA cm^−2^, and fill factor (FF) of 41.76% at the total weight concentration of 10 mg mL^−1^ when dissolved at room temperature. By increasing the dissolution temperature to 50 °C and optimizing the total weight concentration of 11 mg mL^−1^, a higher PCE of up to 12.20% was obtained, with a significantly increased FF of 69.58%. These results suggest that a slightly higher temperature may increase the solubility of BTIC‐4Br and may optimize the morphology of the blend films. After changing to longer alkyl chains, BTIC‐BO‐4Br exhibited a dramatically improved PCE of 14.03%. Noteworthy, the best performance was achieved by BTIC‐2Br‐*m*‐based devices, which showed an outstanding PCE of 16.11% with decreased bromine substitutions (Table S3–S6, Supporting Information). As shown in Table S2, Supporting Information, the influence of the dissolution temperature on BTIC‐BO‐4Br and BTIC‐2Br‐*m* may be negligible if the acceptors had a sufficiently high solubility in the chloroform (CF) and chlorobenzene (CB) solvents. The BTIC‐2Br‐*m*‐based device exhibited a slightly higher *V*_oc_ (0.88 V) compared with that of BTIC‐4Br (0.87 V) due to the higher LUMO energy levels. With a more efficient light harvesting,^[^ 38 ^]^ the *J*_sc_ of BTIC‐2Br‐*m* may be significantly increased, up to 25.03 mA cm^−2^. To the best of our knowledge, the PCE obtained in this study (16.11%) had the highest value among the brominated acceptors in binary PSCs. However, compared to the non‐brominated acceptor Y5 (6.15%, PBDB‐TF as the same donor; Table S10, Supporting Information), the brominated acceptors exhibited a significant potential to pump the efficiency of the non‐fullerene OSCs. The external quantum efficiency (EQE) curves of the three devices have been shown in Figure 2b. The devices based on BTIC‐BO‐4Br and BTIC‐2Br‐*m* exhibited EQEs of over 75% at a range of 500 to 800 nm. Furthermore, the optimal BTIC‐2Br‐*m*‐based device exhibited the most efficient photo‐response, with a maximum value of about 82.5%, while the corresponding internal quantum efficiency (IQE) reached a value of 91.7% (Figure S6, Supporting Information) across the entire length of the wavelength range. The integral current density of the EQE of BTIC‐2Br‐*m*‐based devices was 23.84 mA cm^−2^, which was higher than those of the devices based on other two acceptors (19.68 and 20.21 mA cm^−2^ for BTIC‐4Br, 23.13 mA cm^−2^ for BTIC‐BO‐4Br). These values were in agreement with the *J*_sc_ values (about 5%), indicating that the results about the *J*–*V* curves were highly reliable. To evaluate the high PCE levels of the PBDB‐TF:BTIC‐2Br‐*m*‐based device, one of the optimized inverted devices was sent to Enli Tech Optoelectronic Calibration Laboratory for certification. The device obtained a PCE of 15.04% with a *V*_oc_ of 0.88 V, a *J*_sc_ of 24.24 mA cm^−2^, and an FF of 70.52% (Figure S19, Supporting Information).

**Figure 2:**
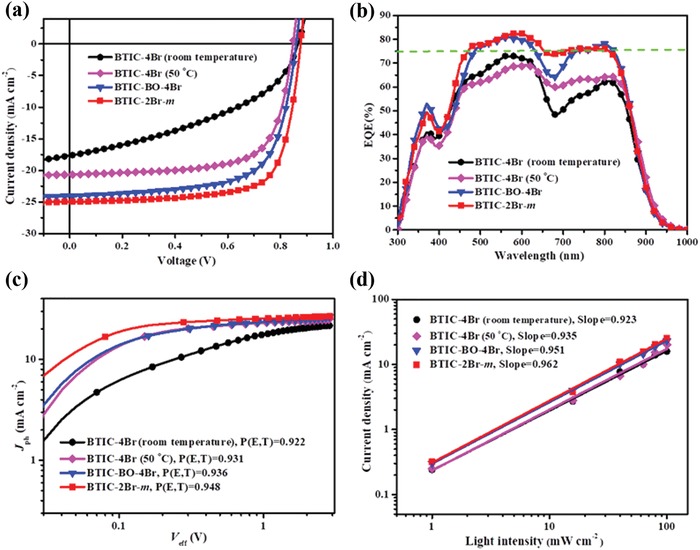
a) *J*–*V* characteristics. b) EQE spectra. c) *J*_ph_ versus *V*_eff_, d) *J*_sc_ versus light intensity of BTIC‐4Br‐, BTIC‐BO‐4Br‐, and BTIC‐2Br‐*m*‐based devices.

**Table 2:** Photovoltaic properties of the optimized PSCs based on PBDB‐TF: acceptors under AM 1.5G, 100 mW cm^−2^ Illumination

Acceptors	*V*_oc_ [V]	*J*_sc_ [mA cm^−2^]	*J*_cal_ [mA cm^−2^]	FF [%]	PCE_max_ (PCE)^c)^ [%]
BTIC‐4Br^a)^	0.87 (0.87 ± 0.01)	20.65 (20.33 ± 0.41)	19.68 (19.43 ± 0.22)	41.76 (41.24 ± 0.55)	7.51 (7.39 ± 0.23)
BTIC‐4Br^b)^	0.85 (0.85 ± 0.01)	20.67 (20.24 ± 0.53)	20.21 (19.79 ± 0.39)	69.58 (69.14 ± 0.47)	12.20 (11.98 ± 0.34)
BTIC‐BO‐4Br^a)^	0.86 (0.86 ± 0.01)	24.06 (23.81 ± 0.25)	23.13 (22.94 ± 0.29)	67.84 (65.45 ± 2.15)	14.03 (13.91 ± 0.18)
BTIC‐2Br‐*m* ^a)^	0.88 (0.88 ± 0.01)	25.03 (24.87 ± 0.27)	23.84 (23.77 ± 0.17)	73.13 (71.31 ± 1.84)	16.11 (15.92 ± 0.21)

^a)^ The mixed solution was dissolved at room temperature

^b)^ The mixed solution was dissolved at 50 °C

^c)^ The average efficiency value was calculated from 15 devices.

To further investigate the effect of bromination on the exciton dissociation of the device based on the non‐fullerene acceptors, the charge dissociation probability (*P* (*E, T*) = *J*_ph_/*J*_sat_) values of the optimized PSCs were calculated by measuring the curves of the photo‐generated current density against the effective voltage.^[^ 49, 50 ^]^ As shown in Figure 2c, the PBDB‐TF:BTIC‐2Br*‐m*‐based device exhibited the highest value (94.8%) for the calculated charge dissociation probability compared to the PBDB‐TF:BTIC‐BO‐4Br‐based device (93.6%) and PBDB‐TF:BTIC‐4Br‐based device (92.2% and 93.1%). This higher *P* (*E, T*) value implies a more efficient exciton dissociation at the PBDB‐TF:BTIC‐2Br*‐m* interface, which is beneficial for increasing the FF and PCE of the BTIC‐2Br*‐m*‐based OSCs to some extent. The charge recombination behavior of the OSCs was assessed to study the relationship between *J*_sc_ and the light intensity (*P*_light_) of the devices,^[^ 51, 52 ^]^ which was defined as *J*_sc_ ∝ *P*_light_^α^. As shown in Figure 2d, the fitted slope values for the PBDB‐TF:BTIC‐4Br, the PBDB‐TF:BTIC‐BO‐4Br, and the PBDB‐TF:BTIC‐2Br‐*m*‐based devices were 92.3% (93.5%), 95.1%, and 96.2%, respectively. The higher value of the PBDB‐TF:BTIC‐2Br‐*m*‐based device demonstrated a lower degree of a bimolecular recombination during the transportation of the charge carrier.

In addition to the exciton dissociation and bimolecular recombination of the devices, the charge carrier transport ability also plays an important role in high‐performance OSCs. The charge carrier mobilities of the blend films were measured using the space‐charge‐limited current (SCLC) model with single‐carrier devices consisting of ITO/ZnO/PBDB‐TF:acceptors/PDINO/Al for the electron mobility and ITO/PEDOT:PSS/PBDB‐TF:acceptors /MoO_3_/Ag for the hole mobility. As plotted in Figure S8, Supporting Information, the devices based on BTIC‐4Br, BTIC‐BO‐4Br, and BTIC‐2Br‐*m* with PBDB‐TF exhibited hole/electron mobilities of 4.8 × 10^−5^/1.1 × 10^−5^ cm^2^ V^−1^ s^−1^, 1.4 × 10^−4^/4.5 × 10^−5^ cm^2^ V^−1^ s^−1^, and 1.9 × 10^−4^/1.1 × 10^−4^ cm^2^ V^−1^ s^−1^, respectively. Compared with the BTIC‐4Br and BTIC‐BO‐4Br devices, the higher hole mobilities and the electron mobilities of BTIC‐2Br‐*m*, in addition to a more balanced μ_h_/*µ*_e_ value, are beneficial for the transportation of charges and the efficiency of carrier collection, which in turn result in an increased FF and *J*_sc_.^[^ 53, 54 ^]^

Subsequently, the measure of photoluminescence (PL) quenching was obtained to confirm the exciton dissociation and the charge‐transfer behavior observed in the blend films. The excitation wavelength of the BTIC‐4Br, BTIC‐BO‐4Br, and BTIC‐2Br‐*m* acceptors was set at 820 nm based on their maximum absorptions. **Figure** **3**a–c shows the PL spectra of BTIC‐4Br, BTIC‐BO‐4Br, and BTIC‐2Br‐*m* and their blend films with PBDB‐TF. The PL emission peaks of the pure acceptors appeared within the range of 850–1100 nm. When mixed with PBDB‐TF, the PL spectra of the blend films was dramatically quenched by 60.3% for BTIC‐4Br, 83.7% for BTIC‐BO‐4Br, and 92.9% for BTIC‐2Br‐*m* at a photoexcitation wavelength of 820 nm. These results indicate that the electron transfer from PBDB‐TF to BTIC‐2Br‐*m* or BTIC‐BO‐4Br was much more efficient than that for BTIC‐4Br, which correlates with the higher *J*_sc_ and FF values found of the corresponding solar cells.^[^ 55 ^]^ Thereafter, we calculated the transient photovoltage (TPV) and transient photocurrent (TPC) of the devices to investigate their dynamics behavior based on the three brominated acceptors. Figure 3d shows the charge carrier lifetime curves of the three brominated acceptor‐based devices in an open circuit. We quantified the early‐stage kinetics using a single exponential decay function. The results suggest that the devices based on PBDB‐TF:BTIC‐BO‐4Br and PBDB‐TF:BTIC‐2Br‐*m* may exhibit a slightly longer charge carrier lifetime τ (1.01 and 1.16 µs) compared to PBDB‐TF:BTIC‐4Br (0.75 µs), which may enhance the transportation of charges and reduce the rate of bimolecular recombination.^[^ 56, 57 ^]^ Figure 3e shows the photocurrent transients caused by a perturbation light pulse. The PBDB‐TF:BTIC‐BO‐4Br‐ and PBDB‐TF:BTIC‐2Br‐*m*‐based devices were found to exhibit a significantly accelerated carrier extraction with a *t*_s_ value of 0.24 µs compared to that of the PBDB‐TF:BTIC‐4Br (0.42 µs). The long carrier lifetime and the low carrier extraction time of the BTIC‐2Br‐*m*‐based devices were the most favorable for charge carrier transport and extraction in the OSCs,^[^ 57, 58 ^]^ while the BTIC‐4Br‐based devices exhibited the lowest τ and *t*_s_ values. BTIC‐2Br‐*m* displayed an optimal phase separation size in the blend films and corresponded to the highest *J*_sc_ and FF values of the OSCs.

**Figure 3:**
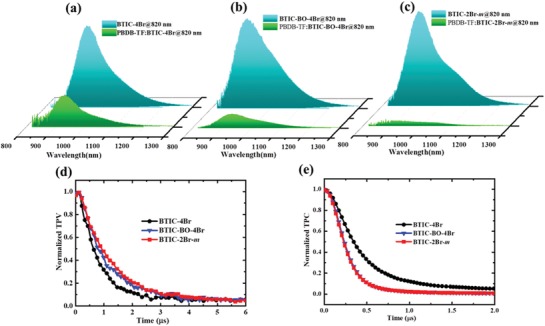
a) The pure BTIC‐4Br (excited at 820 nm), and the blend films of PBDB‐TF: BTIC‐4Br (excited at 820 nm). b) The pure BTIC‐BO‐4Br (excited at 820 nm), and the blend films of PBDB‐TF: BTIC‐BO‐4Br (excited at 820 nm). c) The pure BTIC‐2Br‐*m* (excited at 820 nm), and the blend films of PBDB‐TF: BTIC‐2Br‐*m* (excited at 820 nm). d) Normalized transient photovoltage (TPV). e) Normalized transient photocurrent (TPC) of OSCs base on PBDB‐TF: acceptors.

Appropriate phase separation plays a crucial role in improving the photovoltaic performance of the devices. The morphologies of the three kinds of photoactive layers prepared under optimal conditions were observed under atomic force microscopy (AFM) (**Figure** **4**a–d) and transmission electron microscopy (TEM) (Figure 4e–h). The AFM micrographs showed that the topography of the blend films exhibited the root‐mean‐square roughness values (*R*_q_) of 2.78, 1.56, 1.23, and 1.39 nm, corresponding to the PBDB‐TF:BTIC‐4Br (dissolved at room temperature and 50 °C, respectively), PBDB‐TF:BTIC‐BO‐4Br, and PBDB‐TF:BTIC‐2Br‐*m* blend films. It is noteworthy that excessive aggregations were observed, as shown in Figure 4a,e, which was ascribed to the limited solubility and the strong interaction strength of BTIC‐4Br. When the dissolution temperature was increased to 50 °C, the excessive aggregations were separated and the miscibility of the donor and the acceptor improved, resulting in formation of relatively smaller aggregations (Figure 4b,f). Compared to BTIC‐4Br (Figure 4f), BTIC‐2Br‐*m* (Figure 4h) showed an increase in the degree of phase separation sizes, according to the TEM images. Particularly for the PBDB‐TF:BTIC‐BO‐4Br and PBDB‐TF:BTIC‐2Br‐*m* blend films, interpenetrating networks could be clearly observed compared with PBDB‐TF:BTIC‐4Br. These fiber nanostructures are favorable for efficient exciton diffusion, thus improving the performance of the device.^[^ 59, 60 ^]^ To obtain an in‐depth understanding of the relationship between the structure and performance, a grazing incidence wide‐angle X‐ray scattering (GIWAXS) was used to investigate the molecular orientation and the crystallinity of the three small molecules in the blend films. As shown in Figure 5a–c, the GIWAXS patterns indicated that three blend films exhibited predominantly “face‐on” preferred orientation, as shown by the (100) and (010) diffraction in the in‐plane (*q*_r_) and in the out‐of‐plane (*q*_z_) directions, which favored the promotion of a vertical charge transport in the PSCs.^[^ 61, 62 ^]^ In the blend films, the lamellar (100) peaks of BTIC‐4Br, BTIC‐BO‐4Br, and BTIC‐2Br‐*m* were found in the in‐plane (*q*_r_) at 0.28 Å^−1^ ‐0.30 Å^−1^ (Figure S9, Supporting Information), indicating a similar lamellar stacking in the microstructure of the different blend films. In contrast, the (010) diffraction peaks were located at 1.84, 1.75, and 1.78 Å^−1^, with the corresponding π–π stacking distances of 3.41, 3.59, and 3.53 Å, for BTIC‐4Br, BTIC‐BO‐4Br, and BTIC‐2Br‐*m*, respectively. The difference of the π–π stacking distances between BTIC‐4Br and BTIC‐2Br‐*m* in the blend films implied that the bromine atoms were able to enhance the intermolecular interactions of the small molecules. However, the poor solubility of BTIC‐4Br caused significant difficulties in obtaining the optimal morphology of its corresponding blend film. Although reducing the number of Br atoms and introducing larger side chains may slightly increase the π–π stacking distances in the BTIC‐2Br‐*m* and BTIC‐BO‐4Br blend films, increasing their solubility may result in a favorable BHJ morphology, and may be used to improve their solar conversion efficiencies.

**Figure 4:**
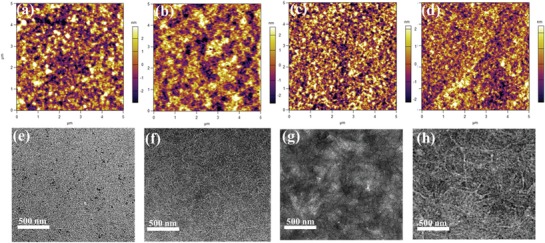
AFM height images (5 × 5 µm) of the blend films: a) PBDB‐TF: BTIC‐4Br (dissolved at room temperature), b) PBDB‐TF: BTIC‐4Br (dissolved at 50 °C), c) PBDB‐TF: BTIC‐BO‐4Br, and d) PBDB‐TF: BTIC‐2Br‐*m*. Bright‐field TEM images of the blend thin films: e) PBDB‐TF: BTIC‐4Br (dissolved at room temperature), f) PBDB‐TF: BTIC‐4Br (dissolved at 50 °C), g) PBDB‐TF: BTIC‐BO‐4Br, and h) PBDB‐TF: BTIC‐2Br‐*m*.

**Figure 5:**
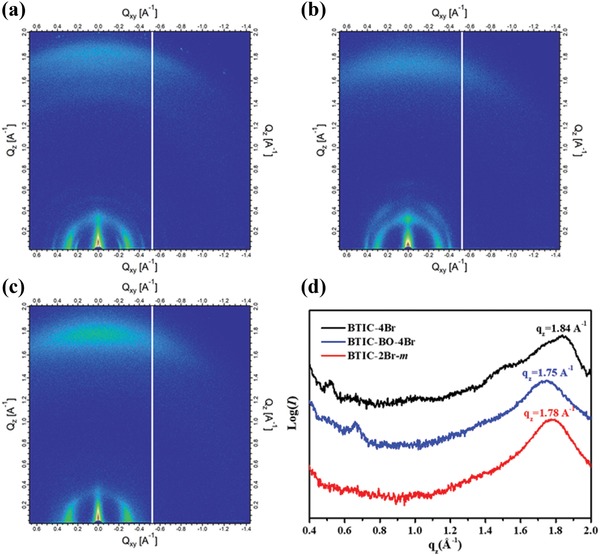
2D GIWAXS patterns of a) PBDB‐TF: BTIC‐4Br, b) PBDB‐TF: BTIC‐BO‐4Br, c) PBDB‐TF: BTIC‐2Br‐*m* blend films, d) GIWAXS linecuts of the out‐of‐plane direction in the PBDB‐TF:acceptors blend films.

In summary, a series of brominated acceptors, namely BTIC‐4Br, BTIC‐BO‐4Br, and BTIC‐2Br‐*m*, were designed and synthesized. The OSCs based on BTIC‐2Br‐*m* exhibited a high PCE (16.11%), corresponding to the highest PCE of brominated end‐capped non‐fullerene acceptors reported for binary OSCs thus far. The high *J*_sc_ (25.03 mA cm^−2^) and the FF (73.13%) values of BTIC‐2Br‐*m*‐based OSCs resulted from a balanced electron/hole mobility, an optimized film morphology, an outstanding quenching efficiency, a high charge dissociation, and a high charge collection efficiency. Hence, the bromination of a non‐fullerene acceptor provides an alternative and promising strategy for achieving high‐performance OSCs. As indicated by the results presented in this work, our message to the organic solar research community is that it will be worthwhile to pay more attention to brominated materials for use in photovoltaic applications.

## Conflict of Interest

The authors declare no conflict of interest.

## Supporting information

Supporting Information
