# Supplementary material for: Bromination: An Alternative Strategy for Non‐Fullerene Small Molecule Acceptors
Source: Adv Sci (Weinh). 2020 Feb 28;7(9):1903784. doi: 10.1002/advs.201903784 (PMC7201261; doi:10.1002/advs.201903784)
Supplement: Supplementary file 1 — Supporting Information [file ADVS-7-1903784-s001.pdf]

Copyright WILEY-VCH Verlag GmbH & Co. KGaA, 69469 Weinheim, Germany, 2020.

Supporting Information

for *Adv. Sci.*, DOI: 10.1002/advs.201903784

Bromination: An Alternative Strategy for Non-Fullerene Small Molecule Acceptors

*Huan Wang, Tao Liu, Jiadong Zhou, Daize Mo, Liang Han, Hanjian Lai, Hui Chen,
Nan Zheng, Yulin Zhu, Zengqi Xie, and Feng He**

Copyright WILEY-VCH Verlag GmbH & Co. KGaA, 69469 Weinheim, Germany, 2020.

Supporting Information

Bromination: An Alternative Strategy for Non-fullerene Small Molecule Acceptors

*Huan Wang, Tao Liu, Jiadong Zhou, Daize Mo, Liang Han, Hanjian Lai, Hui Chen, Nan Zheng, Yulin Zhu, Zengqi Xie and Feng He**

H. Wang, T. Liu, D. Z. Mo, Dr. L. Han, H. J. Lai, Dr. H. Chen, Y. L. Zhu, and Prof. F. He
Shenzhen Grubbs Institute and Department of Chemistry, Southern University of Science and Technology, Shenzhen 518055, China
E-mail: hef@sustech.edu.cn

H. Wang
Faculty of Health Sciences, University of Macau, 999078, Macao, China

J. D. Zhou, Dr. N. Zheng and Prof. Z. Q. Xie
Institute of Polymer Optoelectronic Materials and Devices, State Key Laboratory of Luminescent Materials and Devices, South China University of Technology, Guangzhou 510640, China.

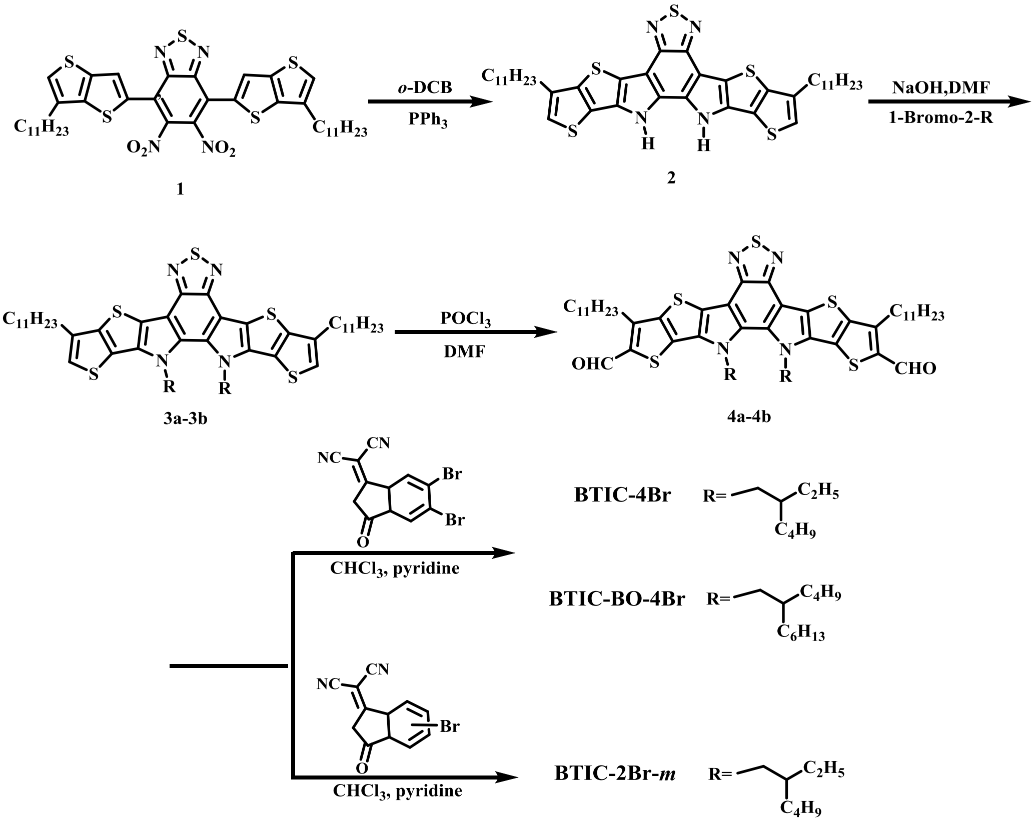

Scheme 1. The Synthetic Routes of the BTIC-4Br, BTIC-BO-4Br and BTIC-2Br-*m*

Synthesis of Compound 2. Compound **1** was synthesized according to reported procedure. [1] To a solution of compound 1 (6.88 g, 8.49 mmol) in o-dichlorobenzene (100 mL) was added PPh_3 (22.20 g, 84.9 mmol) and the mixture was refluxed at 185 °C overnight. After removal of the solvent, the crude product (compound **2**) was obtained as the red solid (7.11 g) and was used directly into the following reaction without further purification.

Synthesis of Compound 3a. A mixture of compound **2** (3.12 g, 4.175 mmol), 5-(bromomethyl)undecane (9.48 g, 38.04 mmol) and sodium hydroxide (1.67 g, 41.75 mmol) was dissolved in 60 mL DMF and refluxed at 90 °C for 15 h. After removal of the solvent, the crude product was purified via column chromatography (silica gel) with petroleum ether/dichloromethane (10/1) as the eluent to yield **3a** as a red solid (0.95 g, 21%).

^{1}H NMR (400 MHz, $CDCl_3$) δ: 7.01 (s, 2H), 4.69 (d, *J* = 8 Hz, 4H), 2.83 (t, *J* = 8 Hz, 4H), 2.10-2.04 (m, 2H), 1.89-1.83 (m, 4H), 1.40-1.27 (m, 68H), 0.90-0.81(m, 30H), 0.68-0.51 (m, 14H).

Synthesis of Compound 4a (BT -BO-CHO**).** To a solution of compound **3a** (0.95 g, 0.87 mmol) in $ClCH_2CH_2Cl$ (30 mL) and DMF (5 mL) at 0 °C, phosphorous oxychloride (2.45 mL, 26.29 mmol) was added slowly under the protection of argon. After stirring at 0 °C for 1 h and then refluxed at 90 °C overnight. The reaction mixture was poured into deionized water (150 mL) and then extracted with dichloromethane three times. The combined organic layer was washed with water, dried over $NaSO_4$, and the solvents were distilled under reduced pressure. The residue was purified by silica gel column chromatograph, using petroleum ether/dichloromethane (1/1, v/v) as the eluent to give **4a** as a red solid (0.7 g, 70.7% yield). ^{1}H NMR (400 MHz, $CDCl_3$) δ: 10.15 (s, 2H), 4.64 (d, *J* = 8 Hz, 4H), 3.21 (t, *J* = 8 Hz, 4H), 2.04 (t, *J* = 4 Hz, 2H), 1.51-1.43 (m, 4H), 1.38 (d, *J* = 8 Hz, 4H), 1.09-0.77 (m, 42H), 0.80-0.59 (m, 14H).

Synthesis of Compound 4b (BT-CHO**).** Synthesis of **4b** was carried out in a similar manner to that of **4a**.

BTIC-4Br. To a solution of BT-CHO (50 mg, 0.049 mmol) in $CHCl_3$ (15 mL) was added 2-(5,6-dibromo-3-oxo-2,3-dihydro-1H-inden-1-ylidene) malononitrile (IC-2Br, 69 mg, 0.196 mmol) and pyridine (0.5 mL) at room temperature. The mixture was heated to reflux for 12 h. After removal of the solvent, the crude product was purified via column chromatography (silica gel) by using $CHCl_3$ as eluent to give BTIC-4Br as a blue-black solid with metallic luster (40 mg, 48%). ^{1}H NMR ($CDCl_3$, 400 MHz, δ/ppm): 9.19 (s, 2H), 8.96 (s, 2H), 8.15 (s, 2H), 4.79-4.77 (d, 4H), 3.27-3.23 (t, 4H), 2.13-2.05 (m, 2H), 1.93-1.85 (m, 4H), 1.52-1.48 (m, 4H), 1.36-1.27 (m, 32H), 1.08-0.99 (m, 12H), 0.89-0.86 (t, 6H), 0.78-0.75 (t, 6H), 0.68-0.65 (t, 6H). MALDI-TOF MS (m/z): 1695.7338 (M^+).

BTIC-BO-4Br. To a solution of BT -BO-CHO (50 mg, 0.044 mmol) in $CHCl_3$ (15 mL) was added 2-(5,6-dibromo-3-oxo-2,3-dihydro-1H-inden-1-ylidene)malononitrile (IC-2Br, 62 mg, 0.176 mmol) and pyridine (0.5 mL) at room temperature. The mixture was heated to reflux for 12 h. After removal of the solvent, the crude product was purified via column

chromatography (silica gel) by using $CHCl_3$ as eluent to give BTIC-BO-4Br as a blue-black solid with metallic luster (46 mg, 58%). ^{1}H NMR ($CDCl_3$, 400 MHz, δ/ppm): 9.18 (s, 2H), 8.94 (s, 2H), 8.12 (s, 2H), 4.78-4.76 (d, 4H), 3.25-3.21 (t, 4H), 2.17-2.09 (m, 2H), 1.92-1.84 (m, 4H), 1.51-1.48 (m, 4H), 1.38-0.64 (m,78H). MALDI-TOF MS (m/z): 1807.8223 (M^+).

BTIC-2Br-*m*. To a solution of BT-CHO (120 mg, 0.117 mmol) in $CHCl_3$ (20 mL) was added IC-Cl-*m* (a mixture of IC-Br-γ and IC-Br-δ, 131 mg, 0.468 mmol) and pyridine (0.5 mL) at room temperature. The mixture was heated to reflux for 12 h. After removal of the solvent, the crude product was purified via column chromatography (silica gel) by using $CHCl_3$ as eluent to give BTIC-2Br-*m* as a blue-black solid with metallic luster (160 mg, 89%). ^{1}H NMR ($CDCl_3$, 400 MHz, δ/ppm): 9.15-9.14 (d, 2H), 8.81 (s, 1.41H), 8.55-8.53 (d, 0.59H), 8.05 (d, 0.54H), 7.89-7.79 (m, 3.58H), 4.80-4.78 (d, 4H), 3.23-3.19 (t, 4H), 2.16-2.10 (m, 2H), 1.91-1.83 (m, 4H), 1.52-1.46 (m, 4H), 1.38-1.20 (m,32H), 1.06-1.00 (m, 12H), 0.88-0.84 (t, 6H), 0.79-0.76 (t, 6H), 0.70-0.65 (t, 6H). MALDI-TOF MS (m/z): 1537.9340 (M^+).

Fabrication and characterization of solar cells device. The inverted device structure was ITO/ZnO/PBDB-TF:Acceptors/MoO_3/Ag. ITO-coated glass substrates were cleaned with deionized water, acetone and isopropyl alcohol for 30 minutes once time and dried in the drying oven at 80 ^{o}C for 12 h before used. The ITO glass was then placed in the UV-ozone for 15 minutes and the sol-gel-derived ZnO film was spin-coated onto the ITO substrate followed by thermal treatment at 200 °C for 30 min and cooled to room temperature under vacuum. A mixture of PBDB-TF/BTIC-4Br (1:1.2 by wt/wt ratio) was dissolved in chloroform solution with the the addition of a small of 1-CN (0.5%, v/v) to obtain 10 mg mL^{-1} of solution at room temperature and 11 mg mL^{-1} of solution at 50 ^{o}C for at least 30 min. The mixture of PBDB-TF: BTIC-BO-4Br and PBDB-TF: BTIC-2Br-*m* (1:1.2 by wt/wt ratio) was dissolved in chloroform solution with the addition of a small of 1-CN (0.5%, v/v) to obtain 10 mg mL^{-1} of solution at room temperature. The active layer was spin-coating at 3000 rpm for 45 s to get

the blend film at the thickness of 90-120 nm. Then the blend film was annealed at 120 °C for 10 min. A 10 nm MoO_3 layer and a 100 nm Ag layer were subsequently evaporated through a shadow mask to define the active area of the devices (0.058 cm^2). The power conversion efficiencies (PCEs) were tested under AM 1.5G irradiation with the intensity of 100 mW cm^{-2} (Enlitech.Inc) which was calibrated by a NREL certified standard silicon cell (4 cm^{-2}). The *J*-*V* curves were recorded with the computer-controlled Keithley 2400 sourcemeter in a dry box under an inert atmosphere. The external quantum efficiency (EQE) and internal quantum efficiency (IQE) spectra were measured using QE-R solar cell spectral response measurement system (Enlitech.Inc). The IQE is evaluated by dividing the EQE by the light absorption of the solar cells as the equation 1[2]

$$\mathrm{IQE}=\frac{\mathrm{EQE}}{\text{Total Absorption}}=\frac{\mathrm{EQE}}{\text{1-Reflection-Transmission}} \quad (1)$$

Charge-carrier mobility measurement. Hole and electron mobility of the acceptors was determined by fitting the dark current to the model of a single carrier SCLC using the device structure ITO/PEDOT:PSS/PBDB-TF: Acceptors/MoO_3/Ag and ITO/ZnO/PBDB-TF: Acceptors/PDINO/Al. The SCLC method is described by the equation[3]

$$J=\frac{9}{8}\varepsilon_0\varepsilon_r\mu_h\frac{V_2}{d_3}$$

Where *J* is the current density, ε_0 is the permittivity of the free space, ε_r is the dielectric constant of the polymers, μ_h is the zero-field mobility, and d is the thickness of the active layer. The current density-voltage (*J*-V) characteristic of hole-only devices was conducted on computer-controlled a Keithley 2400 sourcemeter under dark.

AFM characterization. The film morphology was measured by the atomic Force microscopy (AFM) with the tapping mode from Asylum Reserach. The film samples were prepared followed the fabrication method of the solar cells device.

TEM characterization. Transmission electron microscopy (TEM) images were obtained using a techai F30 instrument at an accelerating voltage of 300 kv. The active layer were spincoated onto PEDOT:PSS. Then the substrate were immersed in the deionized water and the the active layer films were floated. Subsequently, the films were ransferred to a TEM grid.

Absorption spectra measurement: The absorption spectra was tested using a UV-Vis-IR spectrophotometer (Shimadzu, UV3600). The solutions were dissolved in chloroform at a concentration of 10^{-5} mol L^{-1} for the UV-visible spectroscopy measurements. The films were spincoated from the chloroform solution (4-10 mg mL^{-1}) on a quartz substrate.

Cyclic voltametry measurements: Cyclic voltammetry measurements were performed by an electronchemical workstation (CHI600E) to determine the HOMO and LUMO levels of the donor and acceptors. The CV curves were tested under an argon atmosphere in an dichloromethane solution of 0.1 mol L^{-1} tetrabutylammonium hexafluorophosphate(n-Bu_4NPF_6) at a potential scan rate of 100 mV s^{-1} with a Pt wire as the counter electrode and Ag/Ag^+ as the reference electrode. Under the same conditions, the redox potential of $ferrocene/ferrocen^+$ (Fc/Fc^+) is set at 0.044 V with the corresponding energy level of -4.8 eV to vacuum. The HOMO and LUMO levels were calculated by the following equation: $HOMO=-(E_{ox}+4.8-E_{Fc/Fc+})$; $LUMO=-(E_{red}+4.8-E_{Fc/Fc+})$.

Transient photovoltage and photocurrent measurement: The transient photovoltage (TPV) and transient photocurrent (TPC) were performed using performance-all-in-one system(paios). The TPV of the solar cells was measured under open-circuit condition via bias light and the TPC of the solar cells was measured under short-circuit condition without bias light.

Photoluminescence measurement. Photoluminescence (PL) spectra of the blend films were carried out by FLS920 spectro-fluoimeter (Edinburgh Instruments) using 450W xenon lamp. Spectrograph detection used Ge detector with reponse range from 800 to 1700 nm and the data was collected and analyzed by equipped F900 systems software.

GIWAXS characterization. Grazing incident wide-angle X-ray scattering (GIWAXS) measurements were carried out at the Xeuss 2.0 using x-ray source of λ =0.134 nm and a beam size of 0.8◊0.8 mm^{-2}. A Pilatus3R 1M detector was used to capture the scattering patterns.

Table S1. The photovoltaic performance of devices based on PBDB-TF:BTIC-4Br at different total concentration.

Concentrations	V_{oc} [V]	J_{sc} [mA·cm^{-2}]	FF [%]	PCE [%]
8 mg mL^{-1}	0.85	12.18	62.40	6.46
10 mg mL^{-1}	0.86	20.50	41.39	7.29
13 mg mL^{-1}	0.86	16.76	41.19	5.92

Table S2. The photovoltaic performance of devices based on BTIC-BO-4Br and BTIC-2Br-*m* with PBDB-TF as the donor dissolved at 50 ^{o}C.

Acceptor	V_{oc} [V]	J_{sc} [mA·cm^{-2}]	FF [%]	PCE [%]
BTIC-BO-4Br	0.86	24.46	66.17	13.92
BTIC-2Br-*m*	0.89	24.90	72.32	16.01

Table S3. Photovoltaic properties of the PSCs based on PBDB-TF:BTIC-2Br-*m* with different D/A ratio. (0.5 v/v of 1-CN in chloroform was used as the solvent additive and the annealing temperature was set at 120 ^{o}C for 10 min)

D/A Ratio	V_{oc} [V]	J_{sc} [mA·cm^{-2}]	FF [%]	PCE [%]
1.2:1	0.87	25.95	67.71	15.28
1:1	0.88	24.66	72.47	15.70
1:1.2	0.88	25.03	73.13	16.11
1:1.5	0.88	24.76	71.02	15.42

Table S4. Photovoltaic properties of the PSCs based on PBDB-TF:BTIC-2Br-*m* with different annealing temperature. (0.5 v/v of 1-CN in chloroform was used as the solvent additive and the D/A ratio was set 1:1.2)

Annealing [^{o}C]	V_{oc} [V]	J_{sc} [mA·cm^{-2}]	FF [%]	PCE [%]
-	0.91	23.34	70.57	14.94
80	0.89	23.63	71.67	15.12
100	0.88	25.13	70.04	15.54
120	0.88	25.03	73.13	16.11
140	0.87	24.05	72.45	15.22

Table S5. Photovoltaic properties of the PSCs based on PBDB-TF:BTIC-2Br-*m* with different thickness.((0.5 v/v of 1-CN in chloroform was used as the solvent additive, the D/A Ratio was 1:1.2 and the annealing temperature was set at 120 ^{o}C for 10 min)

Thickness [nm]	V_{oc} [V]	J_{sc} [mA·cm^{-2}]	FF [%]	PCE [%]
90	0.88	23.82	73.59	15.40
120	0.88	25.03	73.13	16.11
150	0.88	24.81	71.34	15.51
200	0.87	24.24	70.55	14.86
250	0.89	24.10	61.18	13.16

Table S6. Photovoltaic properties of the PSCs based on PBDB-TF:BTIC-2Br-*m* with different solution.((0.5 v/v of 1-CN in chloroform was used as the solvent additive, the D/A ratio was 1:1.2 and the annealing temperature was set at 120 ^{o}C for 10 min)

Solution	V_{oc} [V]	J_{sc} [mA·cm^{-2}]	FF [%]	PCE [%]
CB	0.82	22.66	63.54	11.75
CF	0.88	25.03	73.13	16.11

Table S7. The hole and electron mobilities of devices based on BTIC-4Br, BTIC-BO-4Br and BTIC-2Br-*m* blended with PBDB-TF.

Acceptor	μ_h [cm^2 V^{-1} s^{-1}]	μ_e [cm^2 V^{-1} s^{-1}]	μ_h/μ_e
BTIC-4Br	4.8×10^{-5}	1.1×10^{-5}	4.36
BTIC-BO-4Br	1.4×10^{-4}	4.5×10^{-5}	3.11
BTIC-2Br-*m*	1.9×10^{-4}	1.1×10^{-4}	1.73

Table S8. The electron mobilities of devices based on neat BTIC-4Br, BTIC-BO-4Br and BTIC-2Br-*m* films

Acceptor	μ_e [cm^2 V^{-1} s^{-1}]
BTIC-4Br	2.3×10^{-5}
BTIC-BO-4Br	8.1×10^{-5}
BTIC-2Br-*m*	2.9×10^{-4}

Table S9. The carrier lifetime (τ) and the determined sweeping out times (t_s) of devices based on based on BTIC-4Br, BTIC-BO-4Br and BTIC-2Br-*m* blended with PBDB-TF.

Acceptor	τ [us]	t_s [us]	τ/t_s
BTIC-4Br	0.75	0.42	1.79
BTIC-BO-4Br	1.01	0.24	4.20
BTIC-2Br-*m*	1.16	0.24	4.83

Table S10. The photovoltaic performance of devices based on PBDB-TF:Y5

Acceptor	V_{oc} [V]	J_{sc} [mA·cm^{-2}]	FF [%]	PCE [%]
Y5	0.93	13.57	48.79	6.15

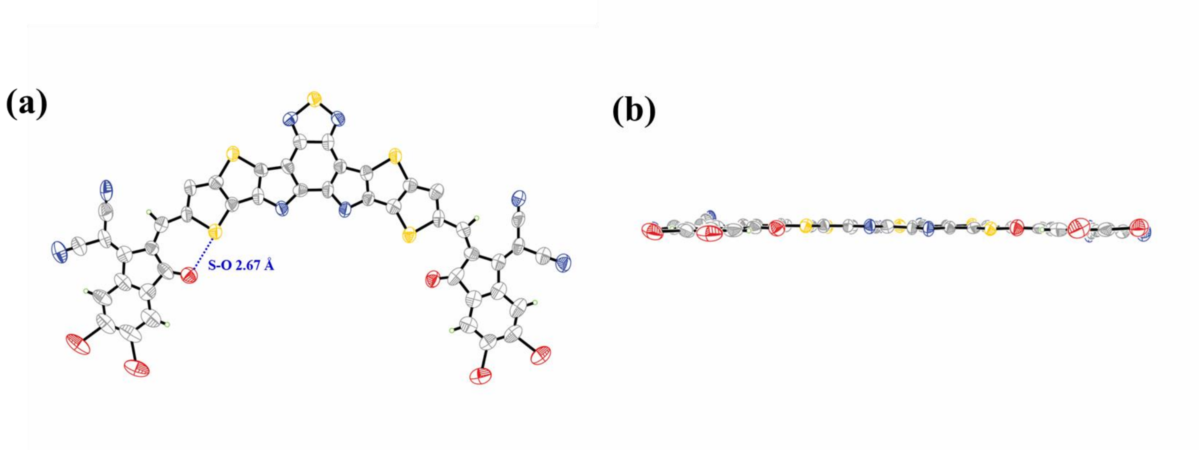

Figure S1. Single crystal structure of BTIC-BO-4Br (2-butyloctyl and n-undecyl side chains were neglected for clarity). (a) Top view. (b) Side view.

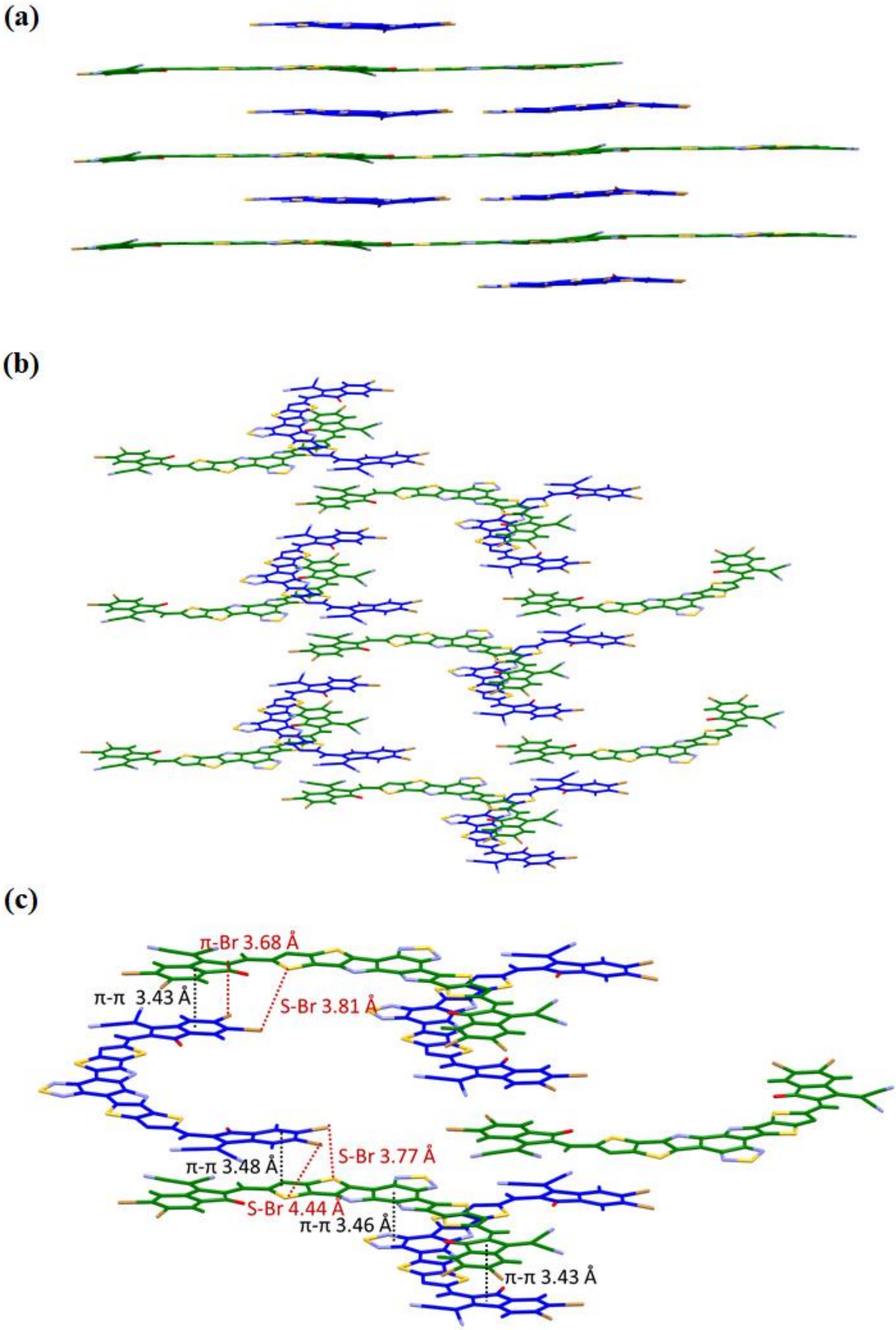

Figure S2. Spiral-like single crystal structure of BTIC-BO-4Br (2-butyloctyl and n-undecyl side chains were neglected for clarity). (a) Side view of adjacent molecules. (b) Top view of adjacent molecules. (c) The interactions between Br···S , Br···π and π ···π of the six molecules in four layers (a closed spiral ring).

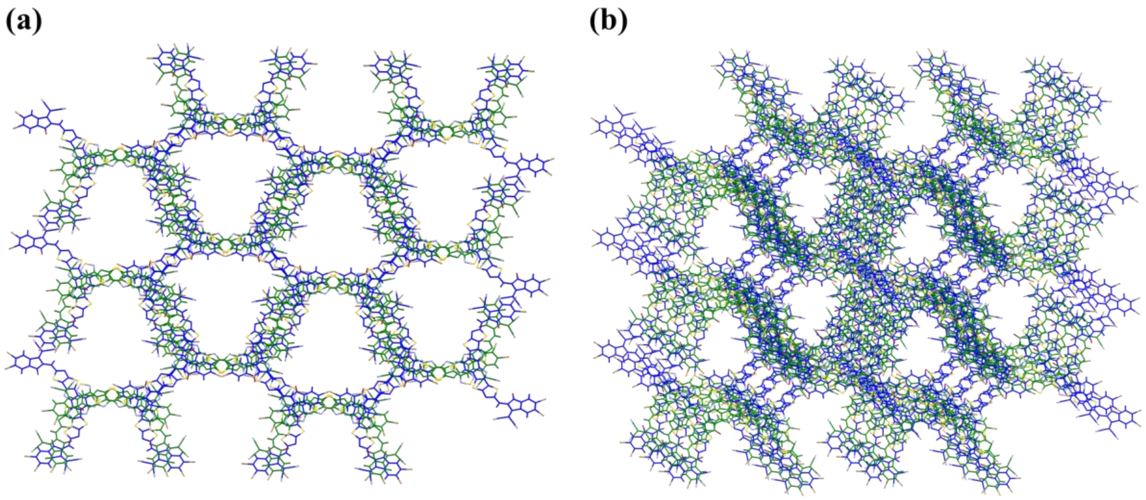

Figure S3. Crystal packing diagrams of BTIC-BO-4Br (2-butyloctyl and n-undecyl side chains were neglected for clarity). (a) Top View. (b) Side view.

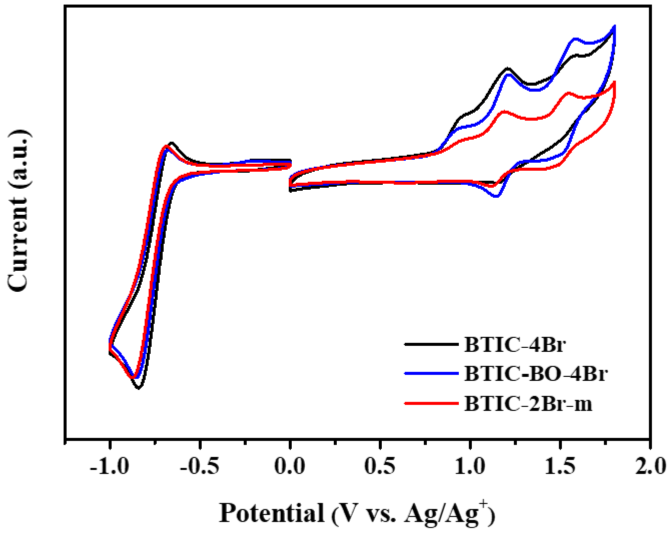

Figure S4. Cyclic voltammogram of the acceptors BTIC-4Br, BTIC-BO-4Br and BTIC-2Br-*m*.

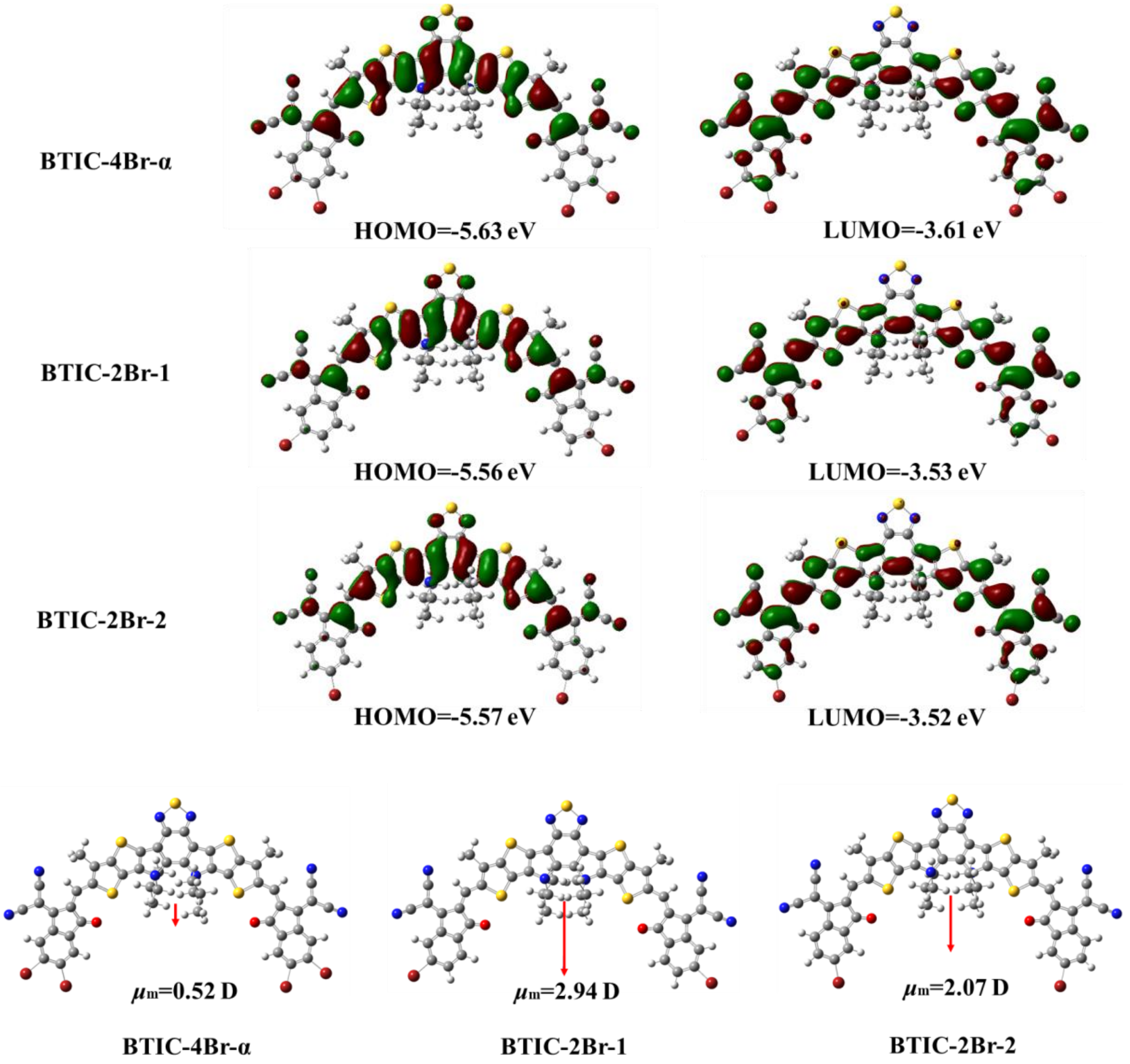

Figure S5. The molecular frontier orbitals and the overall molecular dipole moments of BTIC-4Br-α, BTIC-2Br-1 and BTIC-2Br-2 obtained using DFT calculation.

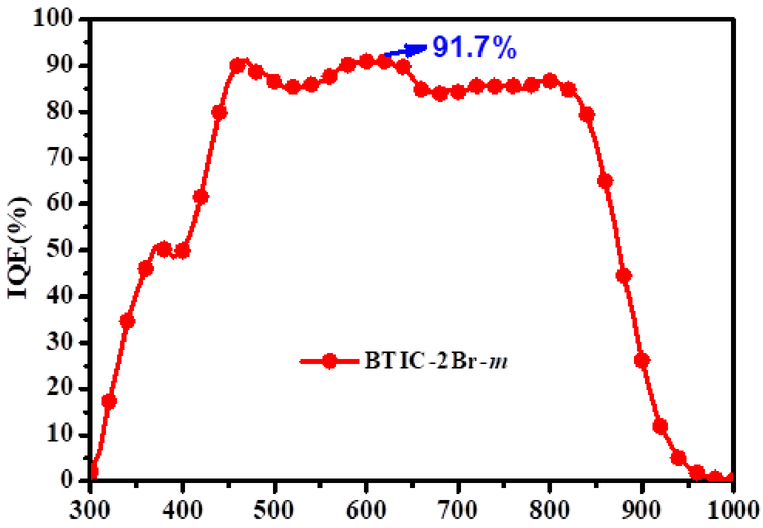

Figure S6. IQE spectrum of the devices based on PBDB-TF:BTIC-2Br-*m*

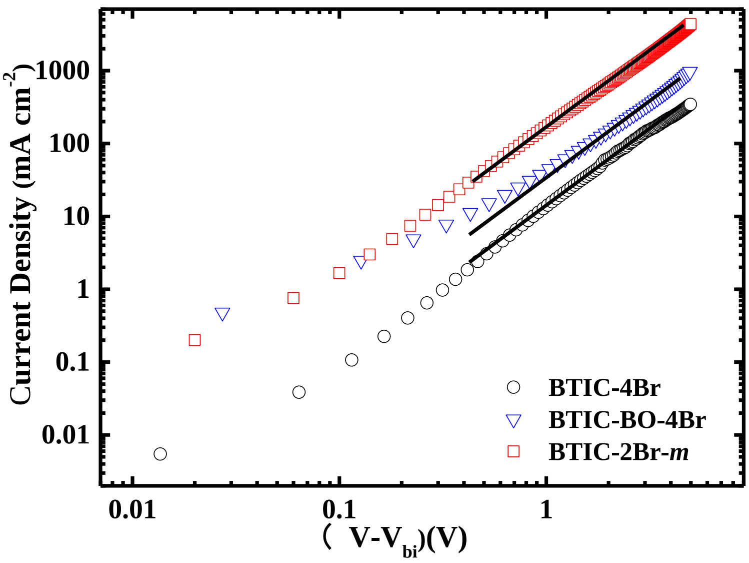

Figure S7. *J*-*V* curves of electron-only devices based on neat BTIC-4Br, BTIC-BO-4Br and BTIC-2Br-*m* in dark.

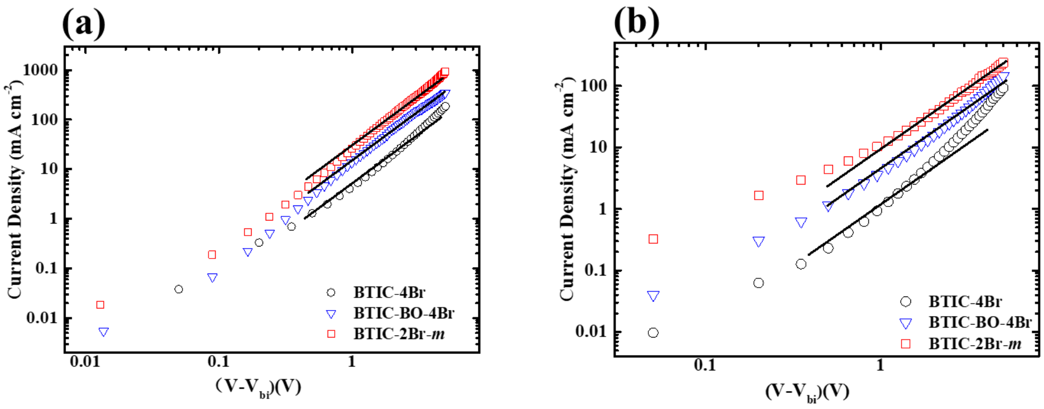

Figure S8. *J-V* curves of hole-only and electron-only devices based on BTIC-4Br, BTIC BO-4Br and BTIC-2Br-*m* blended with PBDB-TF in dark.

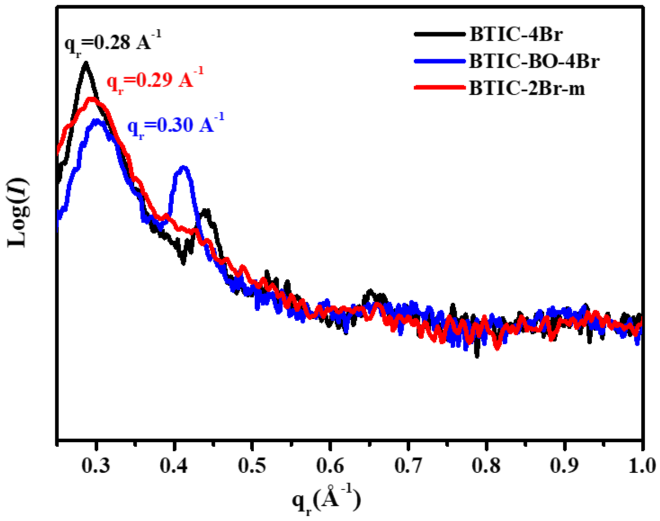

Figure S9. GIWAXS linecuts in the in-plane directions in the PBDB-TF: BTIC-4Br, PBDB-TF: BTIC-BO-4Br and P PBDB-TF: BTIC-2Br-*m* blend films.

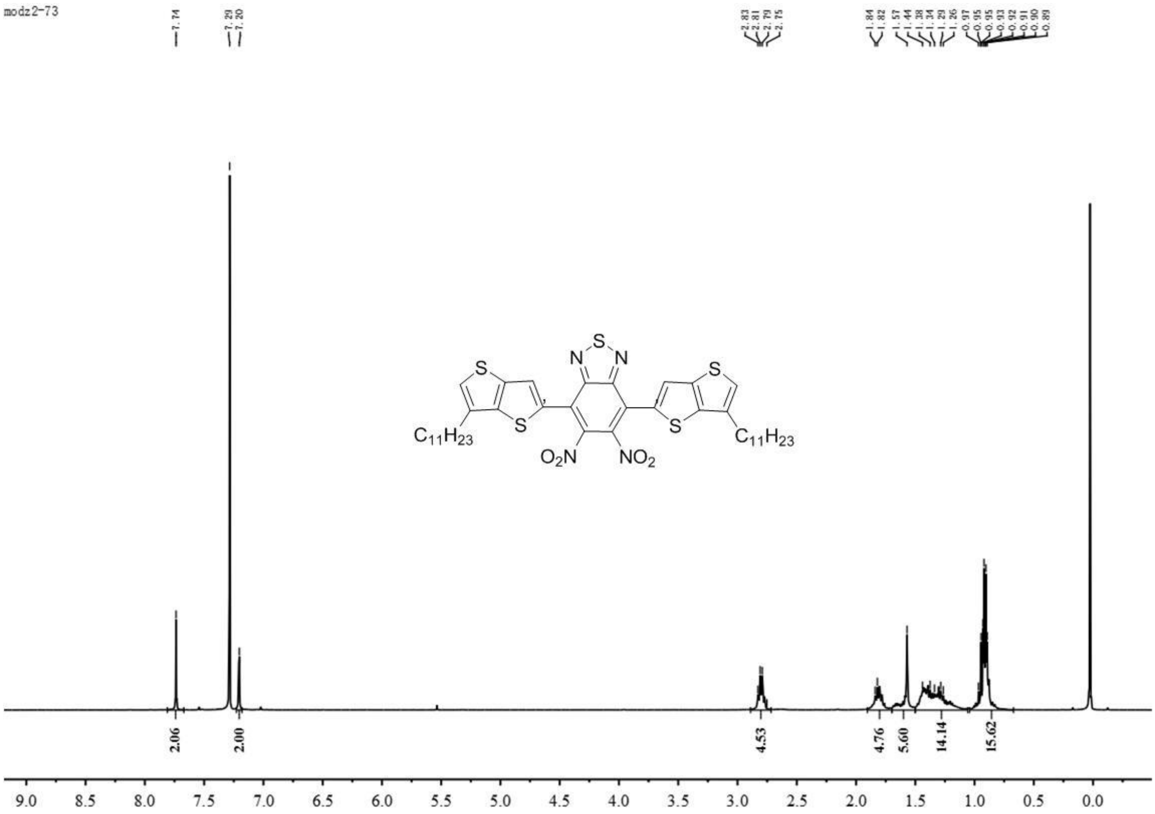

Figure S10. ^{1}H NMR spectrum of Compound **2**.

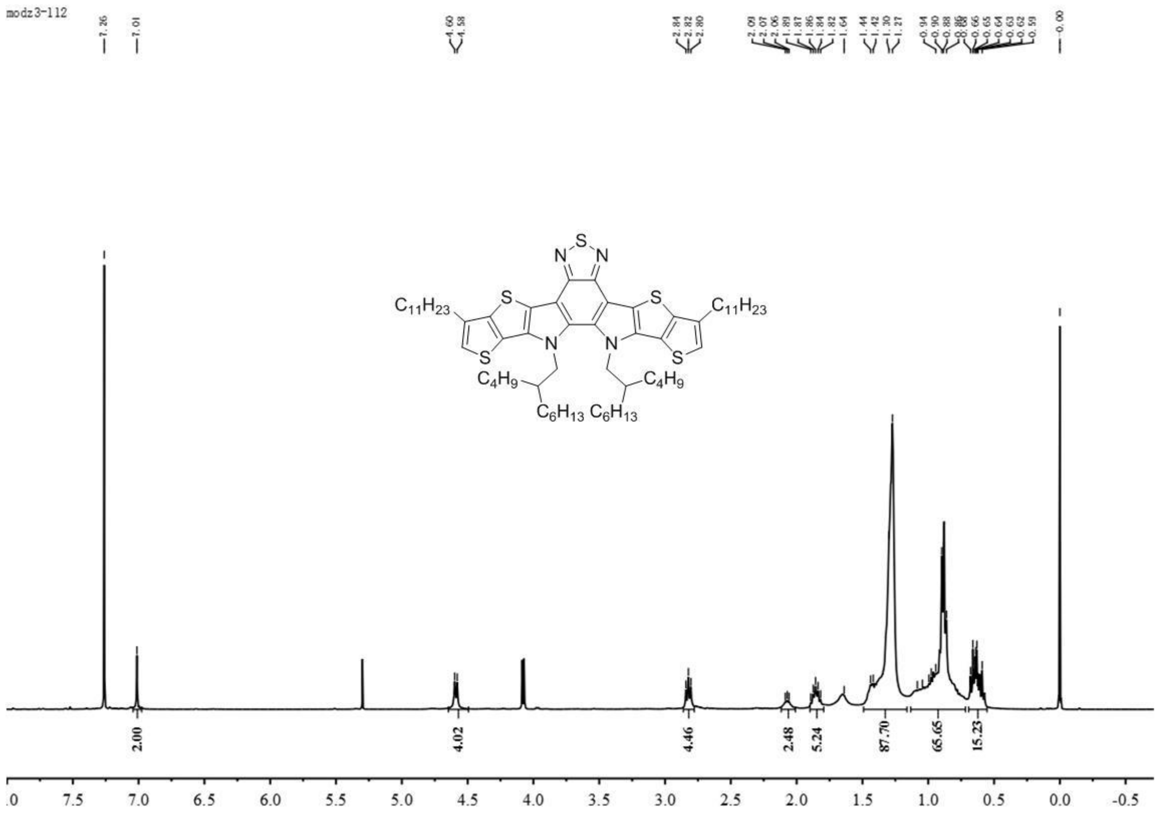

Figure S11. ^{1}H NMR spectrum of Compound **3a**.

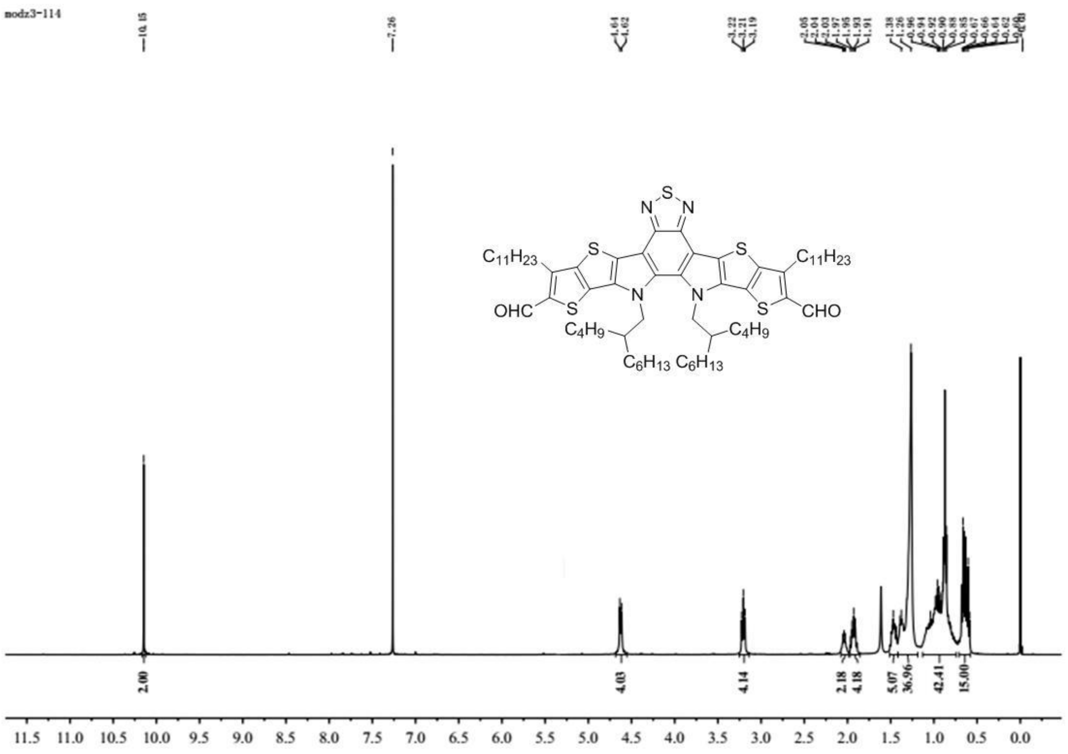

Figure S12. ^{1}H NMR spectrum of Compound BT-BO-CHO.

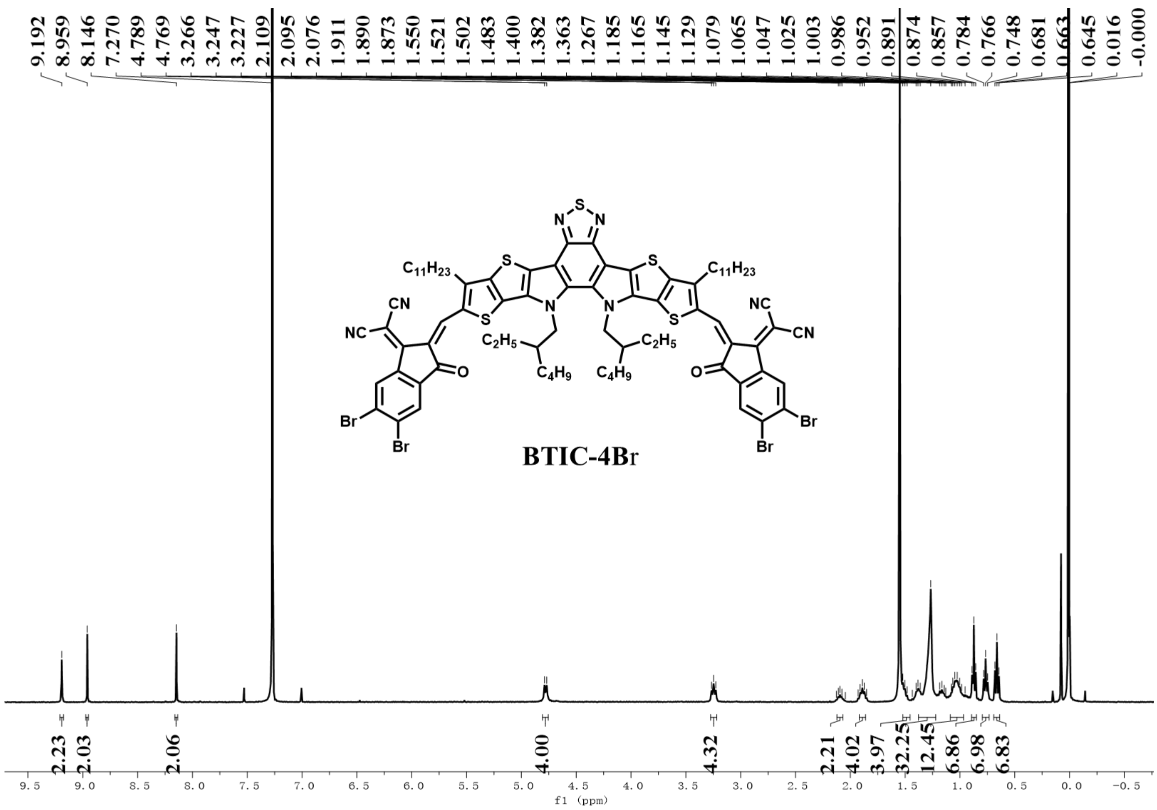

Figure S13. ^{1}H NMR spectrum of small molecule BTIC-4Br.

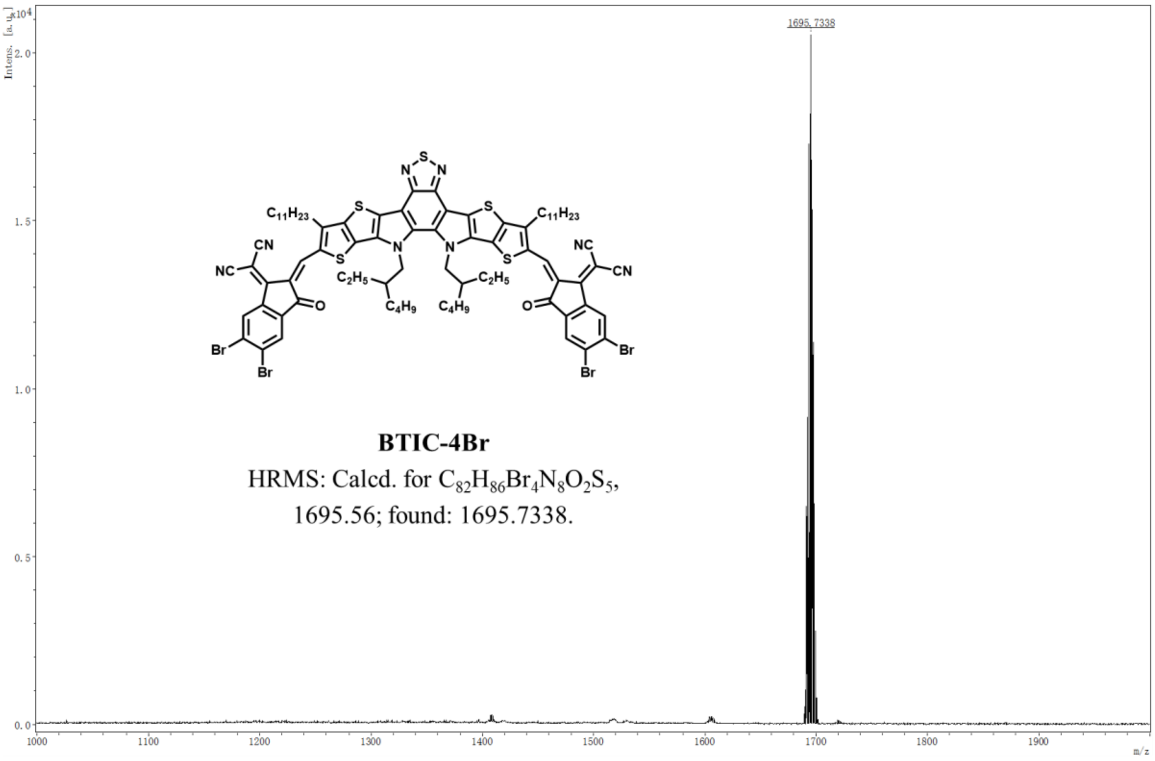

Figure S14. HRMS spectrum of small molecule BTIC-4Br.

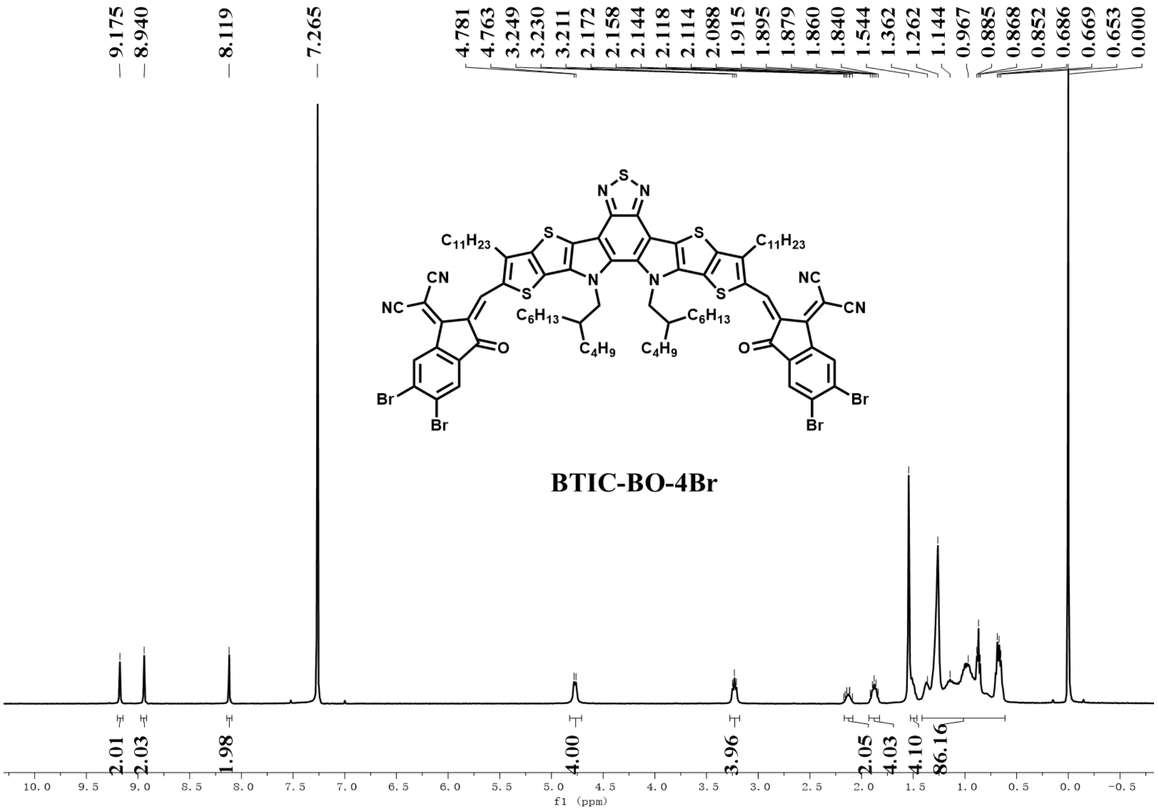

Figure S15. ^{1}H NMR spectrum of small molecule BTIC-BO-4Br.

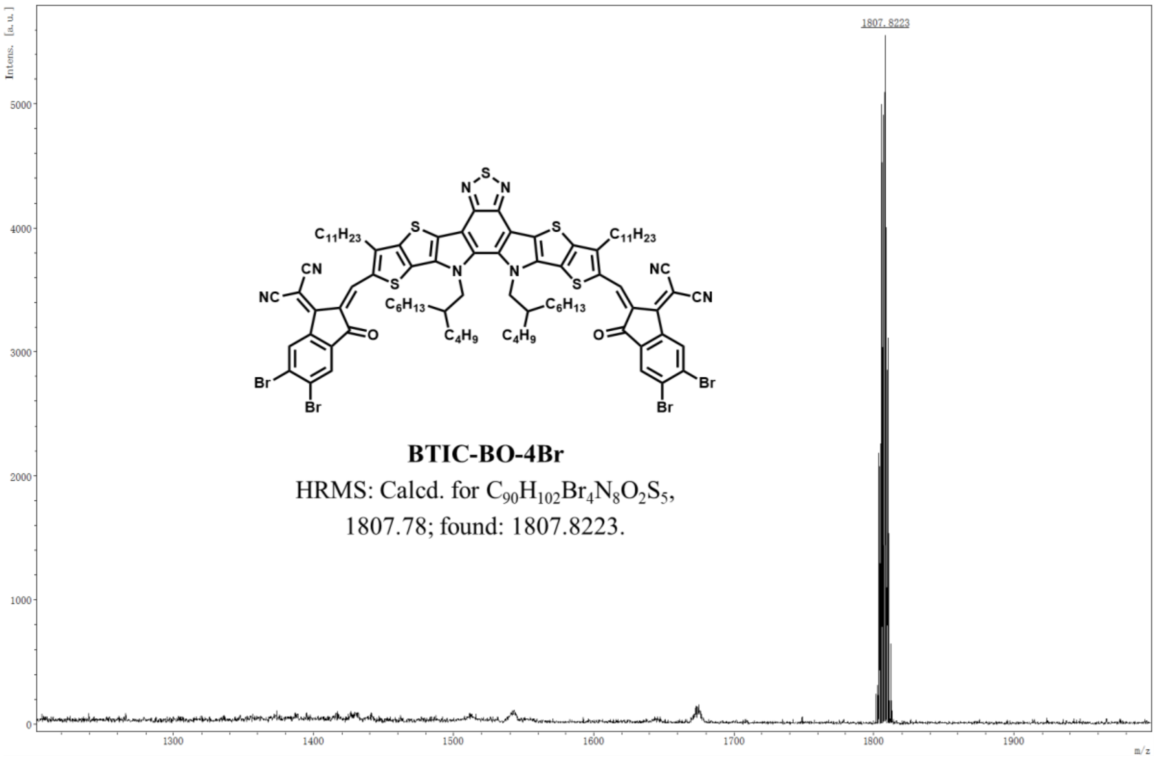

Figure S16. HRMS spectrum of small molecule BTIC-BO-4Br.

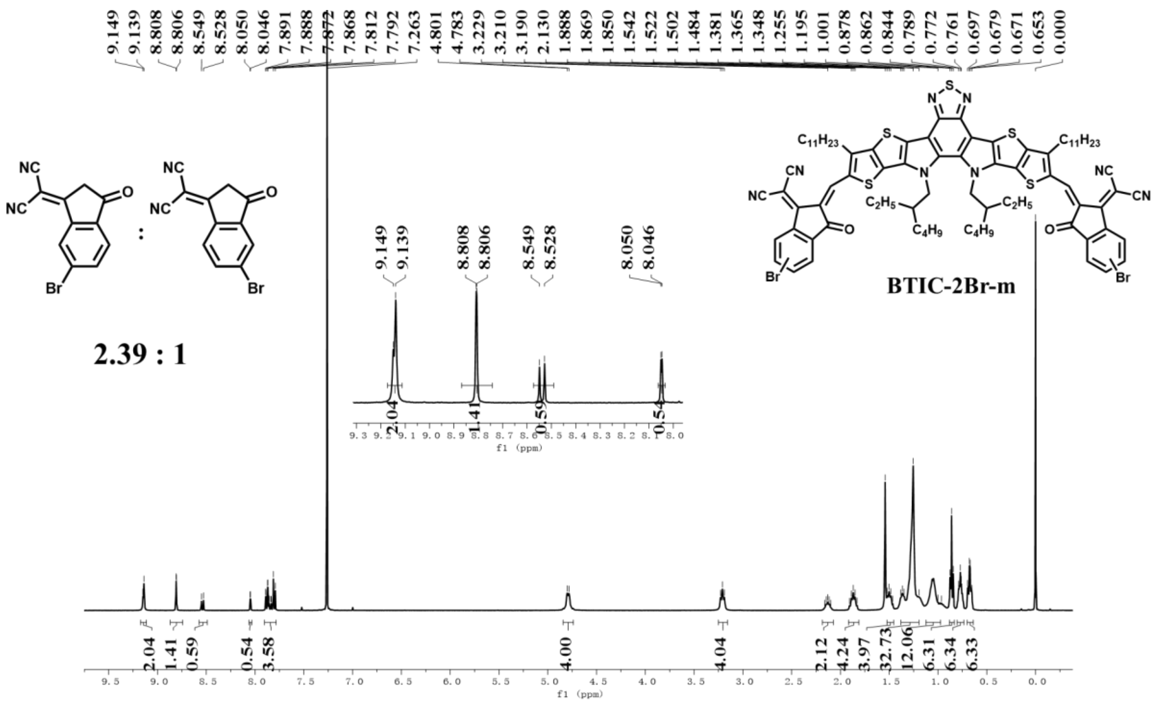

Figure S17. ^{1}H NMR spectrum of small molecule BTIC-2Br-*m*.

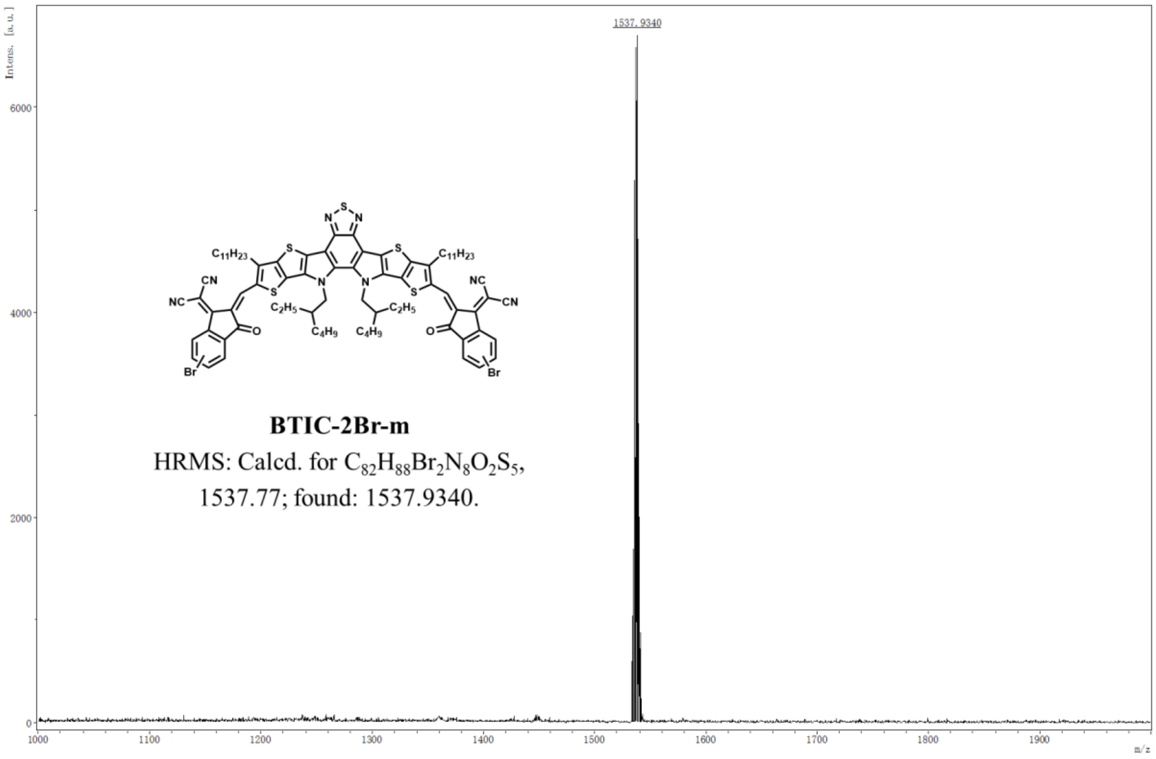

Figure S18. HRMS spectrum of small molecule BTIC-2Br-*m*.

Enlitech

Report No.: PVT191001

Test Report

TAF

Testing Laboratory
3038

**Device Name:**	Photovoltaic Device
**Type:**	BTIC-2Br-m
**Serial No.:**	865-1
**Test Date:**	2019 / 10 / 24
**Customer:**	Southern University of Science and Technology
**Address:**	1088 Xueyuan Avenue, Shenzhen 518055, P.R. China

The test device is measured by the laboratory and the results are given in the content.

The report consists of 4 pages including the cover and is invalid if separated.

The test results of this report are responsible to the device.

The test report should not be reproduced expect in full.

Approved by:

Date: 2019. 10. 28

Enli Tech. Optoelectronic Calibration Lab.
A area, 1F., No. 96, Luke 5th Rd., Kaohsiung, Taiwan, R.O.C

Doc. No. LAB-PV-4-5.10-4C Page 1 of 4 Issued: 2015 / 02 / 02

Enlitech

Report No.: PVT191001

Device information		Environment condition	
Device Name:	Photovoltaic Device	Simulator type	Steady-State Class A
Type:	BTIC-2Br-m	Irradiance:	1000 W/m2
Serial No.:	865-1	DUT Temperature:	25.1 ℃

Test Results and Descriptions

I. Test Results

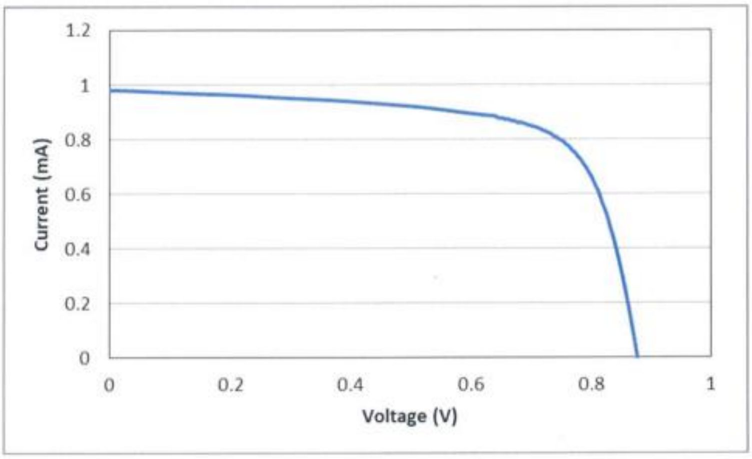

V_{OC}	=	876.10	mV	±	3.51	mV
I_{SC}	=	973.95	μA	±	13.84	μA
P_{MPP}	=	601.77	μW	±	8.85	μW

V_{MPP}	=	728.00	mV
I_{MPP}	=	826.61	μA
FF	=	70.52	%
Efficiency	=	15.04	%
Aperture area	=	3.99	mm^2

Enli Tech. Optoelectronic Calibration Lab.
A area, 1F., No. 96, Luke 5th Rd., Kaohsiung, Taiwan, R.O.C

Enlitech

Report No.: PVT191001

II. Description of the test object

1 Date:

1.1 Date of Receipt: **2019 / 10 / 24**

1.2 Date of Test: **2019 / 10 / 24**

2 Test Site:

Enli Tech. Optoelectronic Calibration Lab.

3 Test Method:

The testing of the sample was performed at Standard Testing Conditions (STC) in accordance with IEC 60904-1:2006 *Photovoltaic devices - Part 1: Measurement of photovoltaic current-voltage characteristics* and *Test standard operation procedure of maximum power measurement of solar cells* [1] under the irradiation with a steady-state class AAA solar simulator according to *IEC 60904-9:2007 Photovoltaic devices - Part 9: Solar simulator performance requirements*. The spectral mismatch is calculated according to IEC 60904-7:2008 *Photovoltaic devices - Part 7: Computation of the spectral mismatch correction for measurements of photovoltaic devices*. The spectrum of the solar simulator is measured with a spectroradiometer. The spectral responsivity (or quantum efficiency) of the device under test is measured with a grating monochromatic according to IEC 60904-8:2014 *Photovoltaic devices - Part 8: Measurement of spectral responsivity of a photovoltaic (PV) device*.

4 Traceability

4-1 Traceability of Reference Cell.

Serial Number:	037-2013
Organization of Calibration:	NREL
Calibration Certificate No.:	1999
Calibration Data:	2019 / 03 / 19
Traceability	NIST

4-2 The traceability of the spectral distribution to SI-Units is achieved by using a calibrated spectroradiometer.

Serial Number:	1811092U1	3011-SP-01512001
Organization of Calibration:	Tai Yi	NIM
Calibration Certificate No.:	K3904240301	GXfs2018-0251
Calibration Data:	2019 / 4 / 29	2018 / 2 / 2

4-3 The traceability of the spectral responsivity to SI-Units is achieved by using a calibrated photo-detector.

Serial Number:	S10-15022
Organization of Calibration:	Gamma Scientific
Calibration Certificate No.:	90064-01
Calibration Data:	2019 / 2 / 19
Traceability	NIST

5 Relative Expanded Uncertainty:

Enlitech Report No.: PVT191001

5.1 Relative expanded uncertainty is estimated based on *Estimated Uncertainty Report of maximum power measurement of solar cells* [2].

5.2 The relative expanded uncertainty resulting of the relative combined standard uncertainty multiplied with a coverage factor k = 2 is specified. It corresponds to a level of confidence of 95 %.

III. Literature

1. *Test standard operation procedure of maximum power measurement of solar cells*, LAB-PV-3-1.4B, Enli Technology Co., Ltd.
2. *Estimated Uncertainty Report of maximum power measurement of solar cells*, LAB-PV-3-5.4-3B, Enli Technology Co., Ltd.

（**Blank**）

Enli Tech. Optoelectronic Calibration Lab.
A area, 1F., No. 96, Luke 5th Rd., Kaohsiung, Taiwan, R.O.C

Doc. No. LAB-PV-4-5.10-4C Page 4 of 4 Issued: 2015 / 02 / 02

Figure S19. Independent certification by Enli Tech. Optoelectronic Calibration Lab. of PBDB-TF:BTIC-2Br-*m* blend film solar cell.

References

[1] J. Yuan, Y. Q. Zhang, L. Y. Zhou, G. C. Zhang, H.-L. Yip, T.-K. Lau, X. H. Lu, C. Zhu, H. J. Peng, P. A. Johnson, M. Leclerc, Y. Cao, J. Ulanski, Y. F. Li, Y. P. Zou, *Joule* **2019**, *3*, 1140.

[2] A. Armin, I. Kassal, P. E. Shaw, M. Hambsch, M. Stolterfoht, D. M. Lyons, J. Li, Z. Shi, P. L. Burn, P. Meredith, *J. Am. Chem. Soc.* **2014**, *136*, 11465.

[3] V. D. Mihailetchi, J. Wildeman, P. W. M. Blom, *Phys. Rev. Lett.* **2005**, *94*, 126602.
